# Apical integration theory provides a neural basis for understanding disorganization and consciousness in the schizophrenias

**DOI:** 10.1016/j.crneur.2026.100165

**Published:** 2026-07-24

**Authors:** Steven M. Silverstein, Timothy Zolnik, Matthew Larkum, Judy L. Thompson

**Affiliations:** a University of Rochester Medical Center, USA; b Charité University of Medicine Berlin, Germany; c Humboldt University, Germany

**Keywords:** Schizophrenia, Disorganization, Psychosis, Apical integration theory, Neuromodulation, Perceptual organization, Cognition, Jung, Bleuler

## Abstract

Recent discoveries involving pyramidal neurons with two zones of input integration (apical and basal), the contextual information they provide, and the neural circuitry and neuromodulation systems in which they are embedded, can contribute to our understanding of disorganized symptoms in schizophrenia, including changes in their expression from the prodromal to chronic stages of the disorder. We demonstrate that the concepts and findings inherent to apical integration theory provide evidence in support of some of the seminal understandings of schizophrenia. In particular, considering the functions of two-point neurons allows for a novel neurobiological understanding of both their role in integrating different sources of information and the impairments in integrative functions that have long been hypothesized to be a defining characteristic of schizophrenia. We demonstrate how the weakening of integrative functions, in multiple domains including perception, cognition, language, behavior, and phenomenology, can be understood as arising from: 1) failures of apical dendrite activity to exert modulatory effects on neuronal output, and 2) reduced activity in the cortico-thalamic networks that support this form of contextual modulation. Later sections address topics such as implications of our view for understanding consciousness in schizophrenia, limitations, and future directions.

## Highlights

• Two-compartment neurons implement context sensitivity in multiple mental functions.
• Reduced apical dendrite drive is a potential mechanism of psychotic disorganization.
• This mechanism accounts for fragmentation in perception, cognition, and thought.
• Apical integration theory extends several influential early theories of schizophrenia.

## Graphical abstract

**Figure:**
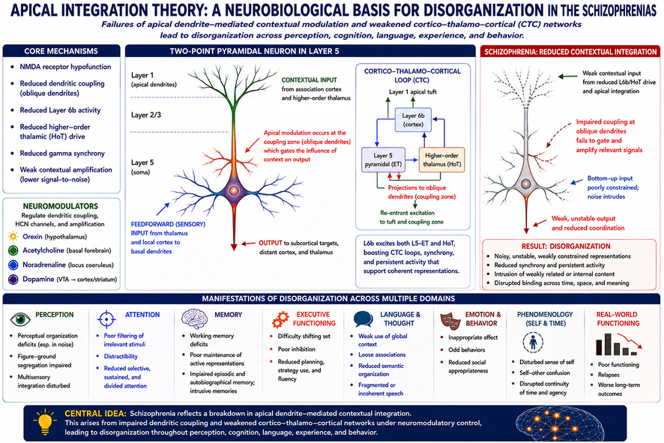

## 1 Introduction: the schizophrenias and the search for unifying neurobiological mechanisms

Schizophrenia is a severe psychiatric condition that affects approximately 0.7% of the population worldwide (McGrath et al., 2008). Multiple genetic and environmental risk factors have been identified, but its pathogenesis remains largely unknown. While it is typically operationalized as a single disorder, it is widely recognized to be a heterogeneous syndrome with an unknown number of subtypes and/or dimensions. One reason for the lack of progress in characterizing heterogeneity (besides the fact that most research studies treat the condition as if it were a single disorder) is that it continues to be diagnosed based primarily on signs and symptoms that are not specific to the condition, that wax and wane over time (and so do not represent trait or vulnerability phenomena), and that have unknown biological bases (Wang and Krystal, 2014). Unlike for most diseases outside of psychiatry, there are no diagnostic or subtype biomarkers for schizophrenia, and many reliable biological findings are found in only a minority of patients (Heinrichs, 2005). This has led to suggestions that we need to, at a minimum, identify biological mechanisms involved in generating individual symptoms, even if it is understood that these may characterize only a subset of those who meet diagnostic criteria for schizophrenia along with others who do not fully meet these criteria. Ideally, it would be possible to identify mechanisms involved in clusters of symptoms and other features (e.g., perceptual and cognitive impairments) that can meaningfully define illness subtypes or dimensions that align with illness course characteristics and treatment response.

To do so, however, requires determining which characteristics of schizophrenia are in fact primary rather than reflective of compensatory processes or consequences of other features. The characteristic symptoms of schizophrenia, as defined by the criteria by which it is currently diagnosed include: psychotic symptoms (also known as *positive* symptoms, so called due to the ‘addition’ of phenomena such as hallucinations and fixed delusional beliefs into consciousness), *negative* symptoms (so called due to the reduction of normative experience), such as restricted facial affect, loss of motivation and drive, and social withdrawal), and *disorganized* symptoms such as reduced coherence in speech, incongruence between ideation and emotion, and abnormal voluntary and involuntary motor behavior.^1^ Although there is a long history in psychiatry of focusing on these symptoms for diagnosis and monitoring of treatment response, it is possible for someone to ‘have schizophrenia’ but, at any given time, have none of these symptoms at threshold level. It is also not clear that all of these symptoms are direct reflections of the condition's primary neurobiological alterations. That is, some symptoms may be compensatory or adaptive responses to the primary neural changes, an idea expressed well over 100 years ago by Bleuler (Bleuler, 1950) with his distinction between primary (emerging from neurobiological impairments) and secondary symptoms, with the latter representing reactions to primary symptoms. In this view, loosening of associations, as typically observed through disorganized speech, was usually considered the most important primary symptom. This referred to a loosening of the associative linkages between mental representations, whether they involve perception, thought, memory, or other cognitive functions. A related contemporary concept was that schizophrenia involves an *abaissement du niveau mentale* (Jung, 1960), which can be translated as a lowering of the level of consciousness, or reduction in psychic tension. This refers to a less differentiated and less organized state of mental functioning (Haule, 1983) characterized by weakening of the normal connections between thoughts, the loss of control of mental contents, an inability to “promote the positive” or “inhibit the negative” (Jung, 1960), the intrusion into consciousness of material that is normally non-conscious and as a consequence, inadequate or inappropriate emotional reactions (Rodriguez, 1983).

The early hypothesis of a lowering of psychic tension in schizophrenia is consistent with Kurt Goldstein's later concept that sufficient excitability in cortical tissue is necessary for the perception of figure-ground relationships (Goldstein, 1939/1995). The latter refers to emergence of a focus of attention during visual perception, and the distinguishing between this focus (typically an object or an event) from the other, background, information that serves as visual context. In Goldstein's view, when brain structure and/or function is compromised, there is a dedifferentiation of the figure-ground process, especially for stimulus fields that are less familiar. This was thought to result in a number of phenomena that have been observed in schizophrenia, such as reduced visual acuity, problems in contour perception, reduced color perception, elevated sensory thresholds, reduced stability in bistable perceptions, and “simplification of the organizational units,” examples of which include reduced perceptual organization and reduced richness of language production (Silverstein, 2016). This idea re-emerged in the 1960s in the work of Paul Meehl, 1962, 1989, who hypothesized that the basis of the integrative impairment that characterizes vulnerability to schizophrenia was a neural alteration he called hypokrisia. He defined this as “a slight quantitative aberration in the synaptic control over the spiking of a neuron” (p. 938) that leads to a dedifferentiated set of neuronal response probabilities, a feature that we discuss below in terms of alterations in the modulatory influence of apical dendrites on the output strength of neurons.

An implication of these seminal views of schizophrenia is that better characterization of reduced integration in brain activity in schizophrenia may lead to a greater understanding of disorganization, which can be seen as a core feature of the condition. It is the most heritable symptom type in schizophrenia (Rietkerk et al., 2008; Legge et al., 2021) and the only symptom dimension significantly associated with the polygenic risk score for schizophrenia (Legge et al., 2021). Disorganization also predicts transition to schizophrenia in people considered to be at high risk for the disorder and in people after a first episode of psychosis (Uzman Ozbek and Alptekin, 2022). It is reliably associated with a cluster of features that have been proposed to identify a clinically meaningful subtype [e.g., more severe cognitive impairment, poor premorbid social and academic functioning, poor current functioning, relapse, functional deterioration, and poor prognosis (Phillips and Silverstein, 2003; Metsanen et al., 2004, 2006; Joseph et al., 2013; Rocca et al., 2018; Pelizza et al., 2023; Wada et al., 2025). Notably, it is more strongly associated with cognitive impairment and poor real-world functioning than are positive symptoms (Ventura et al., 2010). Therefore, clarification of the pathophysiology of disorganization is relevant to the understanding and treatment of the most chronically disabling subtype(s) of schizophrenia. In addition, as discussed by Paliniyappan (Palaniyappan, 2026), directly observable features of psychosis such as disorganization reflect alterations in sensorimotor functioning that can be objectively measured. Thus, grounding the study of the neural underpinnings of psychotic disorders to these signs, as opposed to subjectively reported symptoms such as hallucinations and delusions, has the potential to accelerate progress in neuroscience studies and personalized medicine efforts.

## 2 A neural basis of disorganization in the schizophrenias

**Fig. 1:**
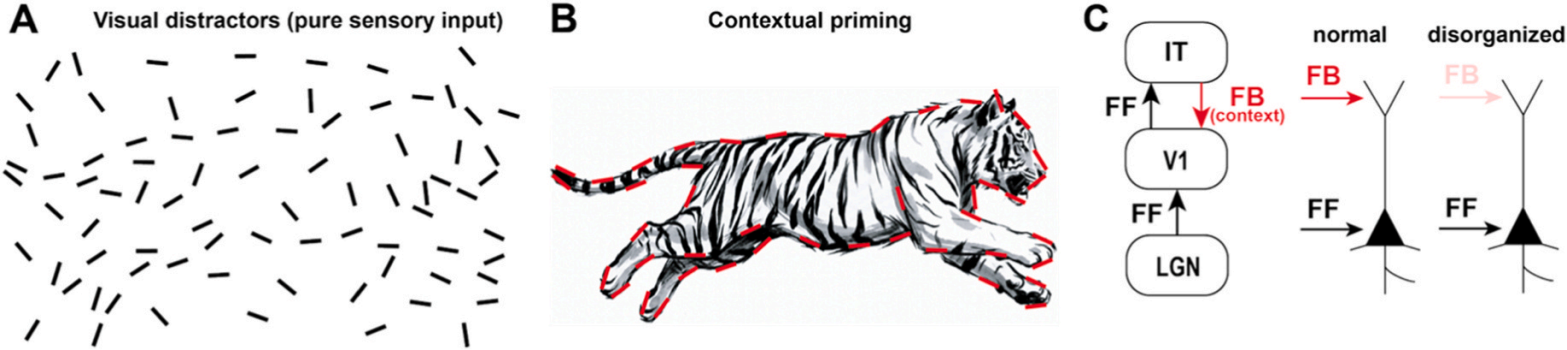
**Contextual coordination versus perceptual disorganization**. (A) Fragmented line segments (visual distractors) represent pure feedforward sensory input arriving in early visual areas without an organizing framework. (B) Top-down prior knowledge (contextual priming) provides a template that guides feature grouping. This allows for rapid perceptual organization of the contour of a tiger in (A) when this is subsequently viewed. (C) In the normal state (left), top-down feedback (FB) from higher-order regions successfully targets the apical dendrites of pyramidal neurons. When this matches feedforward (FF) input, it triggers context-dependent gain amplification, leading to elevated neural response strength and high behavioral detection success (binding). In the disorganized state of schizophrenia (right), impaired apical integration or dendritic decoupling attenuates top-down feedback, reducing context-driven amplification.

In this paper, we argue for an approach that is consistent with the historical views noted above but that is based on a current understanding of the way that neurons cooperatively implement context sensitivity and modulation. This is an update and extension of our prior work proposing a neurobiological and computational basis of a primary binding disturbance in schizophrenia [e.g. (Phillips and Silverstein, 2003; Phillips et al., 2015, 2016),]. We previously proposed (Phillips and Silverstein, 2003, 2013; Phillips et al., 2015; Silverstein and Schenkel, 1997) that impairment in a canonical cortical computation, involving use of contextual information to coordinate neural activity, can account for multiple aspects of schizophrenia, including: reduced organization in perception, thought, language, and motor activity; impairments in working and episodic memory; poor social cognition; and altered representations of self and world. This work was based on evidence from multiple sources, including animal, human, and computational studies (see (Phillips and Singer, 1997) for a review), indicating that there are basic operations common to all or most neocortical regions, such as contextual guidance of learning and processing, and dynamic grouping by synchronized oscillations. In sensory areas, these operations separate a neuron's primary signal (e.g., that coming from its receptive field) from contextual input that modulates the signal salience without altering its content. A paradigmatic example of this distinction can be seen in Fig. 1, where the signal strength of a neuron coding for a visual feature is amplified if the feature is presented within the context of other similar features that together are likely to form a line, edge or surface (i.e., to be part of an object) in the real world. The effects of contextual field input on the processing of receptive field input can be shown to account for a number of perceptual and cognitive functions, including perceptual organization, selective attention, and lexical disambiguation (Phillips and Singer, 1997). More broadly, however, perception, attention, thought and action, learning and memory, and motor functioning all require the amplification of the subset of signals most relevant to the current context and coordination of these signals with ongoing activity in the rest of the brain and body. Outside of sensory systems, where there are no receptive fields per se, it is nevertheless useful to distinguish feedforward signals from contextual input that arises from other streams of processing and that influences the interpretation of feedforward activity. A non-perceptual example of this distinction is how words are understood more quickly when they are presented within a coherent linguistic context (e.g., in a meaningful sentence) (Brothers and Kuperberg, 2021; Brothers et al., 2020, 2023). Evidence that multiple functions of this type are impaired in schizophrenia has been viewed as supportive of the hypothesis of a widespread reduction in the ability of contextual information to coordinate perceptual and cognitive functioning (Phillips and Silverstein, 2003, 2013; Phillips et al., 2015).

A key element in this earlier view was that contextual modulation was implemented via the function of NMDA receptors, given their role in increasing a cell's firing rate when that cell is simultaneously being activated by signals representing relevant contextual information (Phillips and Singer, 1997). By extension, we proposed that the (then) newly discovered feature of NMDA receptor hypofunction in schizophrenia (Javitt and Zukin, 1991; Olney and Farber, 1995; Olney et al., 1999) could account for reduced coordination in perception and cognition and for overt disorganized symptoms, including behavior and emotions that were inappropriate to the current context. More recent work in neurobiology, however, indicates that this perspective is likely too limited. This is in part because: 1) NMDA receptors are colocalized with AMPA receptors on both basal and apical dendritic trees, which does not allow for a separate summing of input from apical dendrites and then using that input as context to modulate basal compartment input; and 2) failures in cognitive coordination of external input cannot explain important aspects of schizophrenia such as hallucinations, since these experiences do not seem to represent responses to external input.

**Fig. 2:**
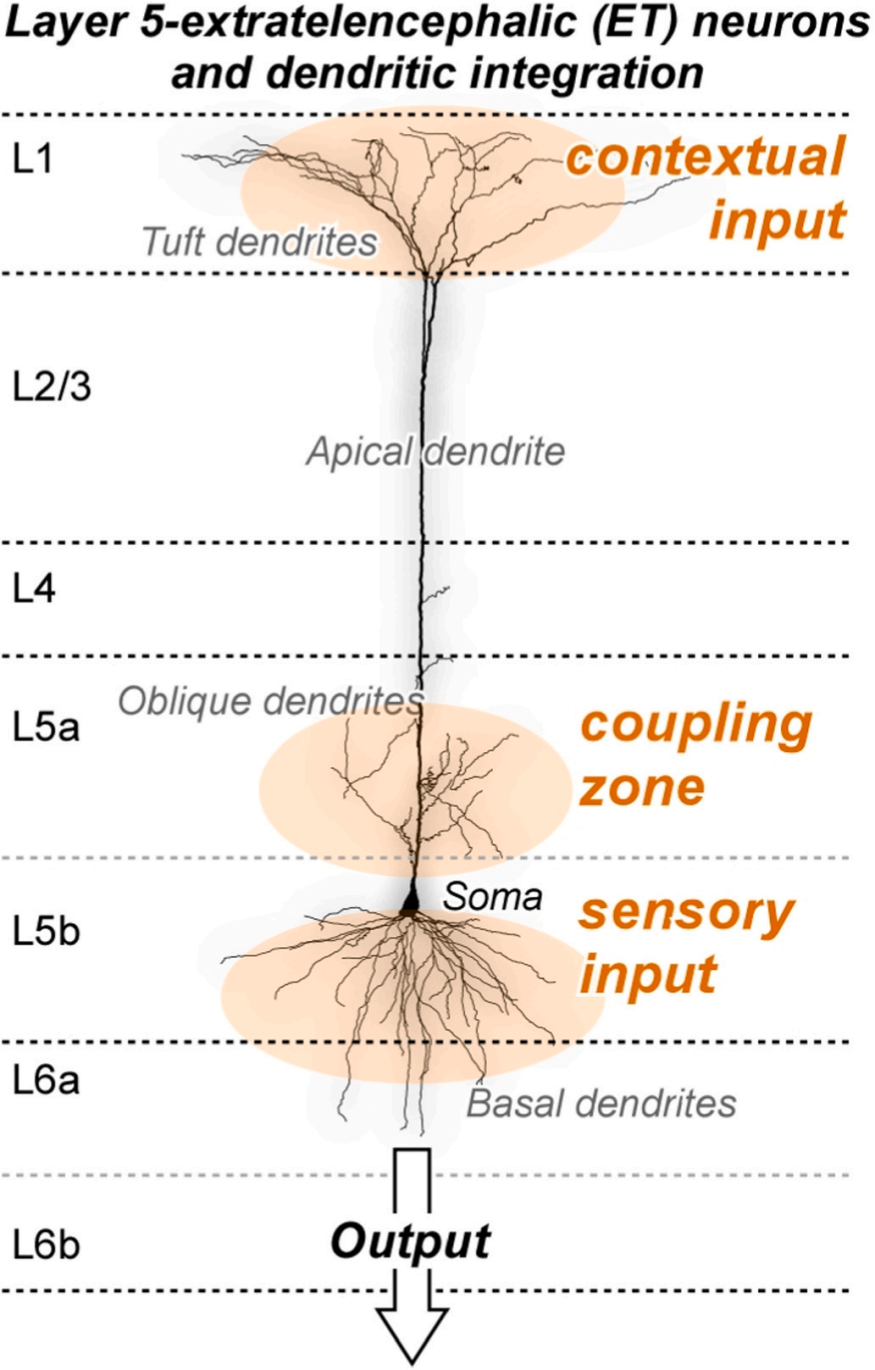
L5-ET neurons integrate three major streams of input across distinct subcompartments. Basal dendrites and soma in layer 5 (L5) receive predominantly feedforward/sensory input, apical tuft dendrites in L1 receive contextual input, and the proximal apical dendrite/oblique region functions as a coupling zone that gates the influence of tuft activity on neuronal output.

Resolution of these challenges to our earlier view were enabled by the discovery of cooperative, two compartment (apical tuft, basal) neurons coupled by the apical trunk (Larkum, 2013; Larkum et al., 1999, 2001; Phillips, 2023). Input to apical dendrites in layer 1 [of layer 5 (L5) and layer 2/3 (L2/3) pyramidal neurons] can be thought of as contextual input in that it originates from regions of the brain outside the receptive field. For example, 90% of L1 input comes from long-range connections from distant cortical regions and includes information about internal state (drives, mood, anxiety) and current goals and expectations (Cauller, 1995; Cauller and Connors, 1994). L5 neurons are the primary output neurons of the cortex and generate some of the largest apical dendritic trees among cortical neurons. They are among the most context-sensitive of all neurons and have the highest number of dendrites in L1 (Moberg and Takahashi, 2022). Consistent with past views of contextual modulation, apical input normally has little effect on the firing rate of a cell unless there is also basal input. However, even weak input to apical dendrites that occurs within a 30ms time window of basal input can significantly strengthen the neuron's output signal (Larkum et al., 2004) (see Fig. 2 below).

An important further distinction between the apical and basal compartments in L5 pyramidal neurons that project primarily to subcortical sites [i.e., extratelencephalic (ET) L5 neurons] is that the basal dendrites in sensory regions receive information from specific (first order) thalamic nuclei (e.g., LGN in the case of vision) that relay sensory information, whereas apical dendrites in L1 receive information from non-specific higher order thalamus (HoT) and from other areas of the brain (including the amygdala) (see Fig. 2). These L5-HoT loops are affected by activity in layer 6b, which is directly linked to multiple arousal mechanisms, as we discuss below. We mention this now only to highlight the multiple sources of input to the two types of dendritic compartments, as well as to note their embeddedness in activity in the brain as a whole.

Studies in behaving rodents demonstrate that disturbances to the contextual drive of the apical dendrites of L5 pyramidal neurons lead to perceptual impairments (Takahashi et al., 2016; Xu et al.). Furthermore, the observation that anesthesia appears to severely diminish the influence of the apical dendritic compartment on the output firing of the neuron (Suzuki and Larkum, 2020) has led to the central hypothesis of Dendritic Integration Theory (DIT), a view similar to apical integration theory. Specifically, it proposes that normal conscious perception in mammals requires the closing of thalamocortical loops via the engagement of apical dendritic drive across a population of L5 pyramidal neurons (Aru et al., 2020; Munn et al., 2023).

Based on these discoveries regarding two-point neurons, an updated hypothesis regarding how NMDA receptor (NMDAR) hypofunction could cause reduced context processing and therefore increased disorganization is that it effectively decouples top-down internal models from the bottom-up sensory stream. Because NMDA receptor-mediated plateau potentials are required for the apical tuft to exert gain control over the cell's output, their dysfunction would sever the “vertical association” that normally allows context to amplify relevant sensory features. Rather than simply shifting dependence toward internal sources via disinhibition, this decoupling would impair the specific circuit mechanism. This suggests that disorganized perception and cognition in schizophrenia could in part arise from the inability of ever-changing contextual information to effectively reach and modulate the primary cortical outputs (Zolnik et al., 2026), leading to an overreliance on more stable, emotion-based contexts to guide mental activity.

It is important to emphasize that NMDA receptors are involved in many non-perceptual functions such as working memory and learning (Collingridge et al., 2013). In addition, NMDA receptor hypofunction in the PFC has been hypothesized to cause weak signaling of contextual cues such as optimal strategies and goals, and inefficient allocation of selective attention (Arnsten et al., 2012). This is relevant for at least three reasons. One is the well-established link between schizophrenia and impairments in PFC function (e.g., executive cognitive function) (Orellana and Slachevsky, 2013). Such deficits would be expected in a condition characterized by hypofunction of NMDA receptors on apical dendrites. Second, our perspective provides a mechanistic framework that can potentially explain impairments as seemingly diverse as those in visual perceptual organization and verbal fluency (a form of executive function). Third, hypofunction in the PFC, including reduced amplification of context-relevant signals, may contribute to the reality testing disturbances that characterize schizophrenia. This is suggested by a recent brain stimulation study (Faerman et al., 2024), which found that inhibition of the PFC increased hypnotizability, or openness to experiencing things as real that would not normally be experienced as real. Hypofunction of the dorsolateral prefrontal cortex has also been observed during dreaming (Muzur et al., 2002), which involves acceptance of non-consensual reality, illogical relationships, and symbolism, and similarities between dreaming and phenomenological aspects of schizophrenia have been previously described (Scarone et al., 2008; Gottesmann, 2006). Data such as these suggest that reduced amplification of context-relevant information streams in the PFC could lead to a number of the disturbances in sense of self and world, and to the emergence of psychotic symptoms that characterize schizophrenia.

In the following subsections, we briefly discuss several key physiological functions that are critical to implementing contextual modulation, some of which have been noted above (e.g., cortical layer 6b contributions). For in-depth discussions of these functions, see (Larkum, 2013; Phillips, 2023; Marvan and Phillips, 2024; Larkum et al., 2009). The remainder of the paper focuses on disturbances in schizophrenia that we propose may be related to alterations in this set of functions. See the graphical abstract for a summary of these points.

### 2.1 Backpropagation-activated-calcium (BAC) spike firing

**Fig. 3:**
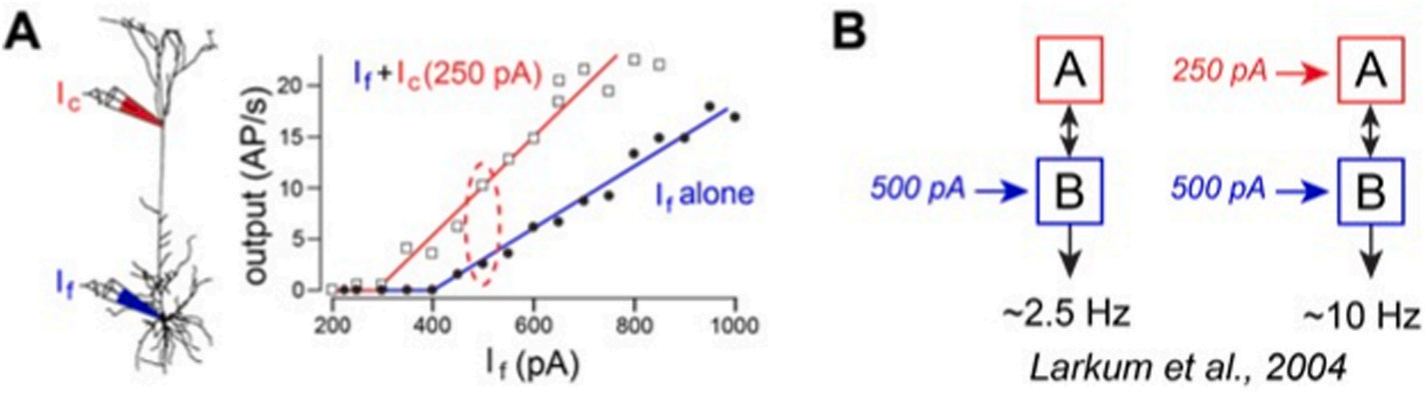
Layer 5 (L5) pyramidal neurons are highly context sensitive due to calcium spike-dependent multiplicative gain modulation. **A)** Experimental configuration showing somatic feedforward current injection (I_f_, blue) paired with distal tuft contextual feedback (I_c_, red). The corresponding plot shows that while feedforward input alone drives a standard threshold-linear output (I_f_ alone; blue curve), the addition of even weak, asynchronous contextual input to the distal apical tuft (I_f_ + I_c_; red curve) significantly amplifies the gain, or slope, of the frequency-current (f-I) relationship. **B)** Schematic representation of this context-driven amplification. Coincidence of somatic/basal input (B) and distal apical tuft input (A) triggers a “vertical association” dendritic calcium conductance. This interaction produces up to a fourfold increase in average output firing rate (shifting from spikes at ∼2.5 Hz to high-frequency bursting at ∼10 Hz, corresponding to the dashed ellipse highlighted in panel A), providing a distinct neural code for stream coincidence. Adapted from (Larkum et al., 2004).

**Fig. 4:**
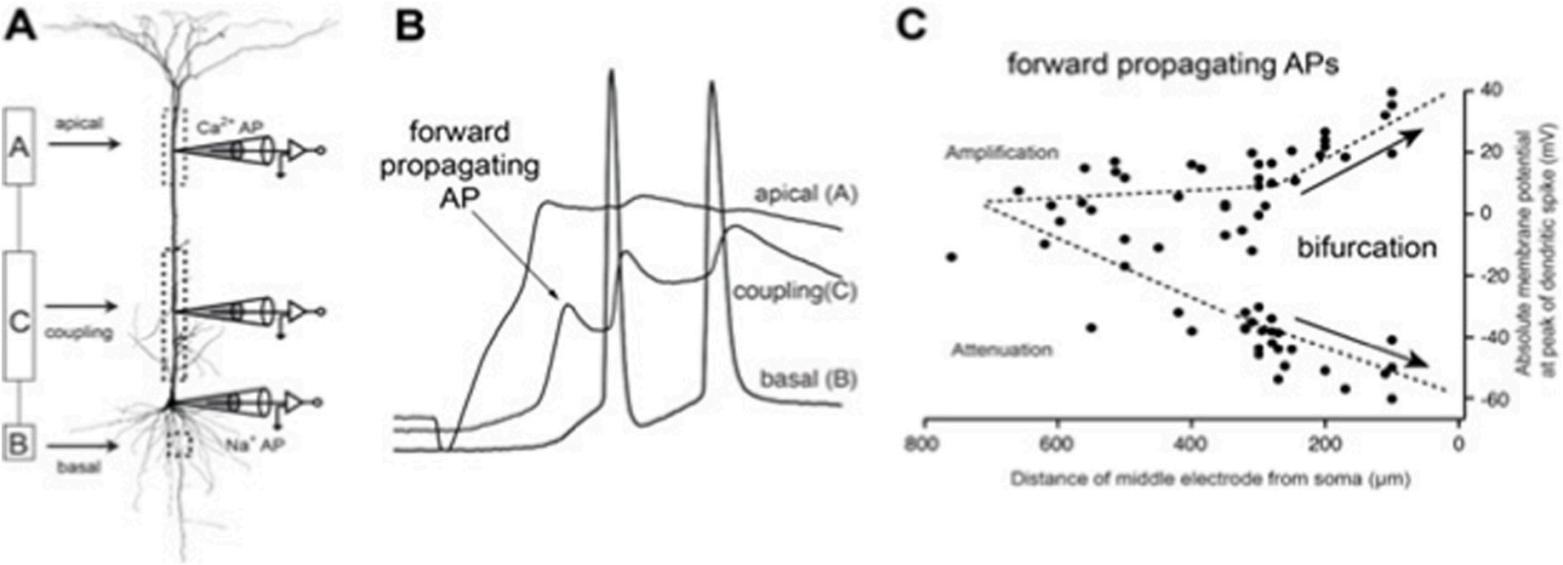
Apical coupling zone. A) Schematic of an L5-ET pyramidal neuron highlighting the three main compartments corresponding to the three main dendritic arborizations receiving synaptic input: the apical tuft (A), the proximal oblique/coupling zone (C), and the basal/somatic region (B). The three recording electrodes at each compartment illustrate the experimental design for testing dendritic coupling *in vitro*. B) Representative recordings with the three electrodes shown in A. C) The amplitude of the "forward propagating AP" (shown in B) as a function of distance from the cell body across many triple recordings. The amplitudes bifurcate at the "coupling zone" (approximately 200–400 μm from the soma) from cell to cell under *in vitro* conditions. The "coupling zone" acts as a critical regulator where apical modulation of somatic activity is either amplified (enabling contextual integration and facilitating perception) or attenuated (e.g. under general anesthesia). Adapted from (Larkum et al., 2001).

After a pyramidal cell initiates an axon potential, an “echo” of that signal backpropagates from the cell body back the apical dendrites. If this signal is met by calcium spikes emerging from the apical integration zone (AIZ), due to simultaneous or very recent input to apical dendrites, there is a strengthening of the signal sent towards the cell body. That is, when there is approximately simultaneous activation (within 30ms) from bottom-up and top-down sources of information, backpropagation-activated calcium spike firing (BAC firing) occurs. This mechanism shifts the neuron's output signal from single spikes to high-frequency bursts and higher rates of firing in general (Larkum et al., 2004) (see Fig. 3). The increase in firing significantly strengthens the neuron's output signal by multiplicatively increasing the gain of its input-output relationship, as illustrated in Fig. 3A and B. Evidence suggests that both the backpropagating signal from the axonal initiation zone and the calcium spikes from the AIZ are driven to threshold predominantly by the recruitment of NMDA spikes in the basal and the apical tuft dendrites respectively (Larkum et al., 2009). *In vitro* experiments show that the influence of apical tuft input on neuronal spike output is highly regulated by a specific region of the proximal apical dendrite which corresponds approximately to the position of the oblique dendrites around the position of cortical layer 5a (Larkum et al., 2001). This process is called “dendritic coupling,” and the control site on the proximal dendrite is called the “coupling zone” (see Fig. 4). When the coupling zone is depolarized, it acts like a gate, enabling the tuft (contextual) input to influence neuronal output to downstream targets, including the HoT (see section 2.5). Of relevance, altered BAC firing in L5 pyramidal cells has been linked to the genetics of schizophrenia in a computational modeling study (Maki-Marttunen et al., 2019).

### 2.2 HCN channels and I_h_

An additional factor determining the effects of apical input is hyperpolarization-activated cyclic nucleotide-gated (HCN) channels. Current flow through these channels is typically referred to as I_h_. HCN channels are partly active near resting (i.e. subthreshold) membrane potentials, with activation increasing at hyperpolarized potentials. Increased activation allows current to “leak” into the cell, preventing excessive hyperpolarization, particularly in the distal dendritic compartment where the density of these channels in cortical pyramidal neurons is highest. At the same time, I_h_ reduces the overall effects of activity at apical dendrites by shortening the decay time of apical signals and decreasing the degree to which inputs from apical tufts are integrated (Berger et al., 2003; Harnett et al., 2015), thereby suppressing accidental or uncoordinated dendritic integration. This accentuates the importance of simultaneous input (i.e., feedforward input accompanied by contextual information) to both compartments of the neuron, a situation that increases apical integration and the likelihood of subsequent calcium spiking towards the soma. As we discuss below, this is relevant to schizophrenia because modulators such as ACh suppress I_h_ (and also potassium conductances). Under some conditions, this suppression of I_h_ by ACh could lessen the dependence of contextual or internally generated input on concomitant basal input and thus reduce its constraint by sensory evidence (Huang et al., 2009). Such under-constrained top-down effects may be relevant to the genesis of hallucinations. HCN-channel regulation in dendritic spines has also been linked to schizophrenia through findings that HCN1 channels co-localize with DISC1- and cAMP-regulating proteins in primate prefrontal cortical spines, together with broader evidence implicating DISC1-related signaling in schizophrenia (Paspalas et al., 2013; Roberts, 2007).

### 2.3 Inhibition and disinhibition of apical dendrites

Activity within apical and basal compartments is also affected by several classes of interneurons, which differentially affect these regions (Phillips, 2023; Marvan and Phillips, 2024; Marvan et al., 2021). Among these interneuron types are: 1) parvalbumin positive (PV) interneurons, which primarily inhibit basal dendrites and other dendrites near the soma, along with more distal apical dendrites (Kawaguchi and Kubota, 1997); 2) somatostatin positive (SST or SOM) interneurons, which inhibit the AIZ and apical branches (Wang et al., 2004; Murayama et al., 2009), and also inhibit interneurons that inhibit the soma, leading to disinhibition of the soma and strengthening of feedforward input; 3) neurogliaform (NGF) interneurons, which also inhibit the AIZ and apical tuft branches (Palmer et al., 2012); and 4) vasoactive intestinal peptide positive (VIP) interneurons, which inhibit other types of inhibitory cells, but have their strongest effects on SST cells, leading to disinhibition of apical dendrites (Pi et al., 2013; Lee et al., 2013). Inhibition of apical dendrites reduces local apical excitability and is reinforced by hyperpolarization-induced activation of HCN channels, which limits dendritic spike initiation and coupling to the soma, reducing contextual effects. Disinhibition of apical dendrites, in contrast, increases pyramidal cell sensitivity to its contextual input.

Schizophrenia is associated with abnormalities in most of these interneuron types. This includes reduced expression of PV interneurons (Kaar et al., 2019) and reduced activity of PV interneurons in the PFC (Toader et al., 2020), and similar alterations in SST interneurons in multiple cortical regions (Van Derveer et al., 2021). A consequence of reduced activity of PV and SST interneurons is disruption to gamma and theta rhythms, respectively (Javitt et al., 2018; Gonzalez-Burgos et al., 2015). In schizophrenia, these alterations are associated with impairments in various forms of contextual modulation (discussed in the next section), such as perceptual organization, mismatch negativity, and working memory (Javitt et al., 2018; Gonzalez-Burgos et al., 2015; Grutzner et al., 2013; Steullet et al., 2017). PV interneuron dysfunction has also been hypothesized to reduce inhibition in semantic networks, an abnormality that is highlighted in a compelling theory of thought disorganization in schizophrenia (Almeida and Radanovic, 2021). Reduced SST activity and the related disinhibition of apical dendrites is thought to lead to distractibility and excessive processing of irrelevant stimuli and context (Dienel et al., 2025). Reduced SST activity would also be expected to lead to greater interference among neural ensembles, as has been shown in the case of intrusive memories in Alzheimer's disease (Almeida, 2024). This is relevant to schizophrenia as well, however, because, as we describe in Section 3, it is characterized by both reduced SST activity and increased intrusions in perception, memory, and language.

We are not aware of any data yet on NGF interneuron structure or function in schizophrenia. There is also little data on VIP interneurons in schizophrenia, although there is evidence that microduplications of a gene related to the expression of VIP receptor 2 (VIPR2) are a risk factor for schizophrenia and autism (Vacic et al., 2011; Firouzabadi et al., 2017). Also, in mice, overexpression of this receptor type leads to reductions in dendrite length and number (Ono et al., 2026), similar to what has been observed in schizophrenia. The net effect of these changes would be reduced integration of local with global activity, reduced synchronization of neural activity, and impairments in the perceptual and cognitive domains reviewed in Section 3 below. It is important to note that not all interneuron types are affected in schizophrenia. In fact, calretinin interneurons, one of the most common interneuron types in primates, appear to be unaffected in this condition (Vid Prkacin et al., 2023).

### 2.4 The role of cortico-thalamic-cortical (CTC) loops

**Fig. 5:**
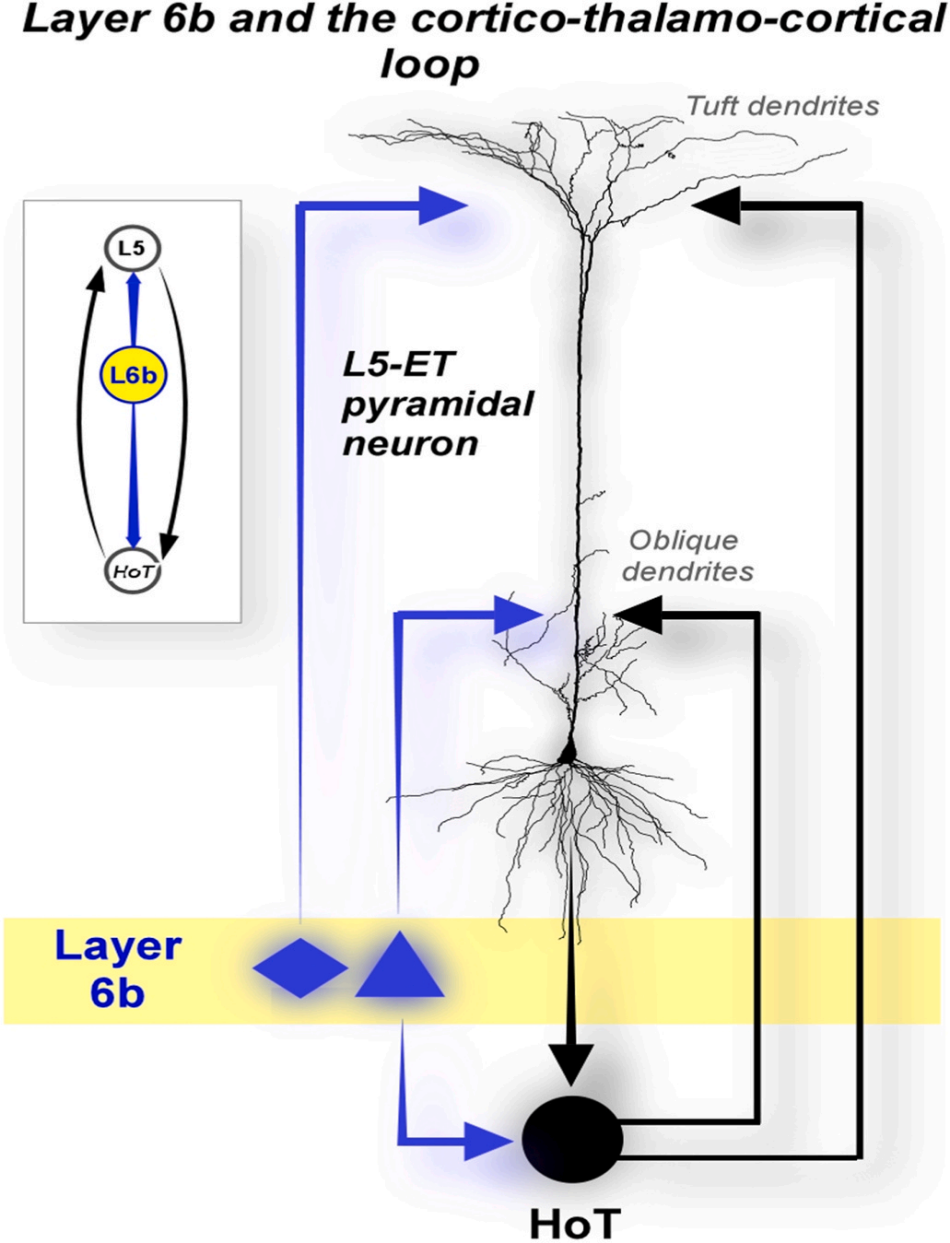
Layer 6b and the cortico-thalamo-cortical loop. Left, simplified circuit depicting layer 6b in the higher-order cortico-thalamo-cortical loop (CTC). Right, Layer 6b neurons (blue) connect to key nodes in the CTC loop (black), positioning them to strongly influence the apical tuft (contextual input) and the proximal apical/oblique dendritic coupling zone. Layer 6b also directly excites higher-order thalamic neurons (HoT), which in turn provide re-entrant excitation to these same sites on the L5-ET neuron. In the context of schizophrenia, reduced L6b and HoT drive would be expected to weaken contextual modulation and destabilize dendritic coupling, thereby reducing the coherence of activity in the loop.

The circuitry discussed above in sections 2.1-2.3 is embedded within a larger set of processes that are coordinated in part by CTC loops. In particular, higher-order thalamocortical loops (Zolnik et al., 2026; Zhou et al., 2011; Gent et al., 2018; Fang et al., 2025) include reciprocal connections between cortical layer 5-ET neurons and neurons in the HoT (see Fig. 5).

These CTC loops may be a larger circuit substrate of perception (Fang et al., 2025). Unlike the regions of the thalamus that serve as sensory relays to the cortex (e.g., the lateral and medial geniculate nuclei), HoT regions relay intracortical information, sending signals from one cortical area back to sensory and association cortices. HoT also provides input to the tuft and coupling zone on L5-ET neurons. HoT contributes to circuits involved in behavioral flexibility and perception, modulating arousal, and integrating and coordinating sources of information. Activity within these loops is modulated by cortical layer 6b neurons, to which we now turn.

### 2.5 The role of cortical layer 6b

Cortical layer 6b, whose functions were relatively unknown until recently, enhances activity in CTC loops (Zolnik et al., 2026). L6b neurons activate both the L5 ET neurons that synapse onto HoT regions, and HoT neurons themselves. To our knowledge, they are the only class of neurons that provide excitatory input to both nodes of CTC loops. Interestingly, both HoT and L6b directly target the coupling zone and the tuft dendrites of L5-ET neurons, a connection which is strongly dependent on NMDA receptors (Zolnik et al., 2024)), suggesting that they may play a critical role in dendritic coupling (Zolnik et al., 2026). L6b is also thought to enhance long-range communication within the cortex by facilitating activity on L5 intratelencephalic (IT) neurons, i.e., L5 neurons that project to cortical sites. Zolnik et al. (2026) proposed that the activating functions of L6b enable strengthening of persistent activity in associated subsets of CTC loops, and that this results in synchronization of firing, sustained attention being directed to the subset of features represented by the amplified loops, and perceptual and other forms of binding [e.g., emergence of figure(s) from ground(s)].

Many forms of binding require sustained activity in neurons. L6b provides a mechanism to enhance and sustain firing of CTC loops. It does this by activating each of the three nodes of these loops: 1) the tufts of L5 apical dendrites in L1; 2) oblique dendrites on the apical trunk, which provides a mechanism for dendritic coupling; and 3) the HoT itself, thereby affecting feedback between HoT and L5 neurons. Reduced input from L6b to the L5-ET tuft and weakened re-entrant output from HoT could reduce contextual input strength (at the apical tuft) and reduce the reliability of dendritic coupling. Together, these alterations can reduce the strength and reliability of the drive on L5-ET neurons, affecting the coherence, stability, and reliability of the CTC loop.

### 2.6 Influences of neuromodulators

The degree of apical amplification is also influenced by mental state via neuromodulators such as orexin (from the hypothalamus), acetylcholine (from the basal forebrain), noradrenaline (from the locus coeruleus), and serotonin (from the dorsal, medial and caudal raphe nuclei) (Phillips, 2023; Avery and Krichmar, 2017). As a result of these inputs, apical dendrites amplify the activity of selected subsets of pyramidal cells. The subset of pyramidal cells whose activities are relevant changes depending on the context or current goals or needs of the organism.

Cholinergic activation increases the effects of apical input on cell output by reducing flow through HCN and K_v_ channels (which normally reduce the influence of apical input by restricting their summation and integration; see above). The density of cholinergic axons and release sites is highest in L1 (Mechawar et al., 2000), where apical dendrites and restrictive ion channels (e.g., HCN and K) are located (Paspalas et al., 2013). These effects on apical dendrite activity operate without affecting responsiveness of the cell soma to its dendritic inputs. Schizophrenia appears to be associated with fewer ACh receptors, and lower levels of ACh are related to psychotic symptoms and cognitive impairments in the disorder (Saint-Georges et al., 2025). Moreover, anticholinergic agents can induce psychotic-like experiences in healthy subjects (Veselinovic et al., 2015) and in people with schizophrenia (Veselinovic et al., 2015; Wojtalik et al., 2012; Schreiber et al., 2018). In addition, recent clinical trials of pro-cholinergic agents have shown promising results in reducing positive and other symptoms of schizophrenia (Brannan et al., 2021; Krystal et al., 2022; Yohn et al., 2022; Sauder et al., 2022), especially the combination of the brain-penetrant muscarinic (M1/M4) agonist xanomeline and the peripheral blocker trospium. Data such as these are consistent with the proposal that psychosis may involve a weakening of adaptive contextual effects and a reduction in signal-to-noise ratios, leading to the formation of novel “figures” based on and maintained by strong internal context.

On the other hand, other data suggest that the downregulation of muscarinic ACh receptors in schizophrenia could be a consequence of abnormally high levels of extracellular ACh, especially during acute psychotic episodes (Sarter et al., 2005). This is consistent with Phillips's (Phillips, 2023) hypothesis that acute psychosis represents transient periods of apical drive (see below) superimposed upon longer periods of reduced context processing. Long-term exposure to agents that increase cholinergic activity have been linked to psychotic symptoms, as shown by the emergence of psychosis following chronic exposure to cholinesterase inhibitors, due to pesticide exposure (Gershon and Shaw, 1961; Bowers et al., 1964). More recent evidence of cholinergic dysregulation in psychosis comes from a study by Weinstein et al. (2024), which demonstrated that in participants with schizophrenia, psychotic symptom severity and working memory deficits were strongly associated with vesicular ACh transporter availability in several cortical and subcortical regions, as assessed with PET and [18F]-VAT.

Like ACh, noradrenaline (NA) promotes wakefulness and enhances the effects of apical depolarization and thus the effects of context (internal and external). However, when NA levels are extremely high, apical drive may occur, leading to contextual field information being fed forward in addition to, or instead of, it having only modulatory effects. Given that activity of cells in the locus coeruleus is regulated by the PFC, current goals and plans can affect the degree of context sensitivity at any given time via their effect on NA levels. Disorganization, cognitive impairment, and negative symptoms have been associated with reduced NA in schizophrenia (Yamamoto and Hornykiewicz, 2004), whereas positive symptoms have been linked to increased NA (Pelegrino et al., 2023). This is consistent with Friston's hypothesis (Friston, 1999) that schizophrenia involves a failure of neuromodulation that leads to impairments in contextualizing sensory input. Further evidence for NA dysregulation in schizophrenia comes from results obtained with various experimental paradigms indicating reduced activation of the autonomic nervous system (suggesting reduced NA activity) during task performance in patients (Maki-Marttunen et al., 2020). An important distinction between NA and ACh is that the effects of NA are more diffuse than those of ACh. As a result, the cholinergic system (which enhances both basal and apical influences on the cell's output) is more involved with enhancing local processing, while the adrenergic system (which primarily enhances apical influences) is more involved with integration of large-scale activity. On the other hand, high levels of NA may increase (rather than decrease, as per its usual effects) I_h_, thereby reducing context sensitivity (Pelegrino et al., 2023).

The neuropeptide orexin (hypocretin) is one of the strongest arousal-promoting molecules in the brain and plays a major role in determining level of wakefulness (Sakurai et al., 2021). In addition to its direct effects, it causes the release of other arousal-inducing modulators such as NA (Jennings and De Lecea, 2019). Cortical layer 6 is the only cortical layer that is sensitive to orexin (Bayer et al., 2004), and the L6b effects noted above are strongly driven by orexin. In addition, L6b activity is increased by the other neuromodulators whose release is stimulated by orexin (Feldmeyer, 2023). Moreover, orexin blocks flow through HCN channels via activation of phospholipase C and protein kinase C (PKC) pathways (Li et al., 2010), providing an additional mechanism by which context sensitivity is increased by orexin. As reviewed in Zolnik et al. (2026), L6b receives long-range cortical feedback from distant cortical regions, especially higher-order frontal and parietal regions (Zolnik et al., 2020). This input to L6b is possible because the axons of cells originating in these regions travel through the white matter that contains spiny dendrites of L6 neurons (Zolnik et al., 2020; Marx and Feldmeyer, 2013). Thus, L6b can receive long-range input from axons that project to other destinations, which can assist with appropriate regulation of CTC loops. However, this depends on the level of neuromodulated arousal. When arousal is low, L6b will likely have little sustained effect on CTC loops. Thus, it can be seen that through their effects on modulators such as NA and input to L6b, signals related to goals and expectations from regions such as the PFC can critically determine the degree to which CTC loops are activated and in this way shape the specific neural ensembles that emerge as figure from ground.

The close link between arousal, attention, and goal activation is consistent with Kraepelin's (Kraepelin, 1919/1971) seminal proposal that the attention deficit prominent in schizophrenia is strongly tied to the impairments in motivation that characterize the condition (Zec, 1995). Studies of orexin and symptoms in schizophrenia have produced mixed results, however. For example, one study demonstrated reduced plasma orexin levels in patients (Lu et al., 2021), and, consistent with this, another study found that higher orexin A levels were related to fewer negative and disorganized symptoms in a subgroup of patients (Chien et al., 2015). On the other hand, a meta-analysis of studies of plasma orexin-A levels in schizophrenia found no clear evidence of abnormalities (Li et al., 2022). However, it has been shown that chronic treatment with antipsychotic medication is associated with increased plasma orexin (Chen et al., 2022). More research is needed to better understand potential orexin alterations in schizophrenia and their links with symptoms, as well as relationships between orexin levels and performance on putative laboratory indicators of cooperative context sensitivity such as perceptual organization.

Serotonin (5-hydroxytryptamine, or 5-HT) has differential effects at different types of 5-HT receptors, of which 16 have been identified (Di Giovanni et al., 2008). We focus here primarily on the 5-HT2A class of serotonin receptor because these are densely expressed on apical dendrites of L5 pyramidal cells. Activation of 5-HT2A receptors has excitatory effects at apical dendrites. It also opposes I_h_ current and therefore increases effects of apical dendrites on cell output. Excessive activity at 5-HT2A receptors has been implicated in the genesis of positive symptoms (e.g., hallucinations (Abi-Dargham, 2007; Leptourgos et al., 2020; Silverstein and Lai, 2021)). In addition, it has been proposed that excessive activity at 5-HT2A receptors leads to a cascade of effects resulting in an increase in positive and negative symptoms (Kim, 2021). A specific link between 5-HT alterations and disorganization symptoms specifically, however, has not been documented.

In contrast to the other neuromodulators mentioned above, dopamine (DA) may, via its modulation of HCN channels, reduce the effects of apical input and increase the weighting of feedforward signals (Arnsten et al., 2012). It is still not clear how DA affects apical integration in L5-ET neurons per se, but it has been shown that these effects are receptor-, cell type- and state-dependent (Lafourcade et al., 2022; Rindner et al., 2022; Mease and Gonzalez, 2021). DA is the most investigated neurotransmitter with regard to schizophrenia. The DA hypothesis of schizophrenia has evolved over the past decades (Howes and Kapur, 2009; Zhao et al., 2025), with current evidence suggesting elevated DA activity in the striatum but decreased activity in extrastriatal and cortical regions, including the PFC (Kegeles et al., 2010; Slifstein et al., 2015; McCutcheon et al., 2019; Brugger et al., 2020). A pathological reduction in cortical DA transmission would be expected to reduce I_h_ current activity and to facilitate and even abnormally enhance apical dendritic influence on processing, but data consistently indicate that schizophrenia is characterized by reduced context sensitivity including on tasks involving PFC control. For example, it is well established that working memory, which is dependent on PFC DA (D'Ardenne et al., 2012), is impaired in schizophrenia (see section 3.4 below). Relatedly, a PET study that examined cortical DA release in schizophrenia demonstrated that reduced PFC DA release was related to reduced fMRI BOLD responses during a WM task (Slifstein et al., 2015). On the other hand, the effects of reduced cortical DA could include increased sensitivity to longstanding emotion-based internal contexts, and may thereby be related to the genesis of delusions and hallucinations.

Furthermore, it may be the case that the DA dysregulation that characterizes schizophrenia contributes to an increase in noisy neural activity, via decreased phasic DA responses for relevant stimuli and increased spontaneous phasic DA release (see (Maia and Frank, 2017)). Such alterations may lead to the formation of novel associations and meanings that are under-constrained by context. For example, such a process may be facilitated by the effects of excess striatal DA, including aberrant salience and perceptual, attentional, and motivational biases towards normally irrelevant stimuli (Olney et al., 2018). This would presumably increase the influence of internal models of the world that are not adequately constrained by relevant external contextual information and could thus lead to disorganized and psychotic symptoms, along with poor performance on tests of external context processing.

A clinical example of this hypothesized process comes from Matussek (1952), in which a patient recounted the subjective experience of reduced perceptual organization as well as one of its consequences, first stating when describing a scene: “I only saw fragments: a few people, a kiosk, a house. To be quite correct, I cannot say that I see all of that, because the objects seemed altered from the usual. They did not stand together in an overall context, and I saw them as meaningless details,” and then noting that “Out of these perceptions came the absolute awareness that my ability to see connections had been multiplied many times over.” While such experiences may be fleeting, some “insights” may persist if they trigger or are supported by strong emotional reactions or pre-existing associative networks (e.g., organized around traumatic memories). Jung called these patterns of associations ‘feeling-toned complexes,’ and viewed them as functioning as semi-autonomous psychic elements that could intrude into consciousness, causing symptoms such as intrusive thoughts, unexpected emotions, and other experiences of self-disturbance. This concept is similar to the modern concept of ‘parasitic foci’ (Hoffman and McGlashan, 1993), which refers to attractor states that form independently of the statistical structure of the world and that reinforce themselves. It is thought that the persistence of such states is enabled in part by “cortical decoupling,” a concept that overlaps with those of reduced cooperative context sensitivity and reduced dendritic coupling.

In sum, the activity of neuromodulators affects the extent to which top-down influences in the form of apical dendrite activity are integrated with basal compartment input to implement contextual modulation. In schizophrenia, there are alterations in multiple components of these systems. These appear to have the effect of reducing modulatory effects overall, while at the same time allowing for pockets of apical drive that can become decoupled from feedforward brain activity and dynamic changes in contextual information. This scenario would result in both disorganization and positive symptoms that become progressively strengthened by internal models and increasingly disconnected from external sources of information over time.

## 3 Schizophrenia-related impairments that suggest alterations in apical dendrite function and associated mechanisms

**Fig. 6:**
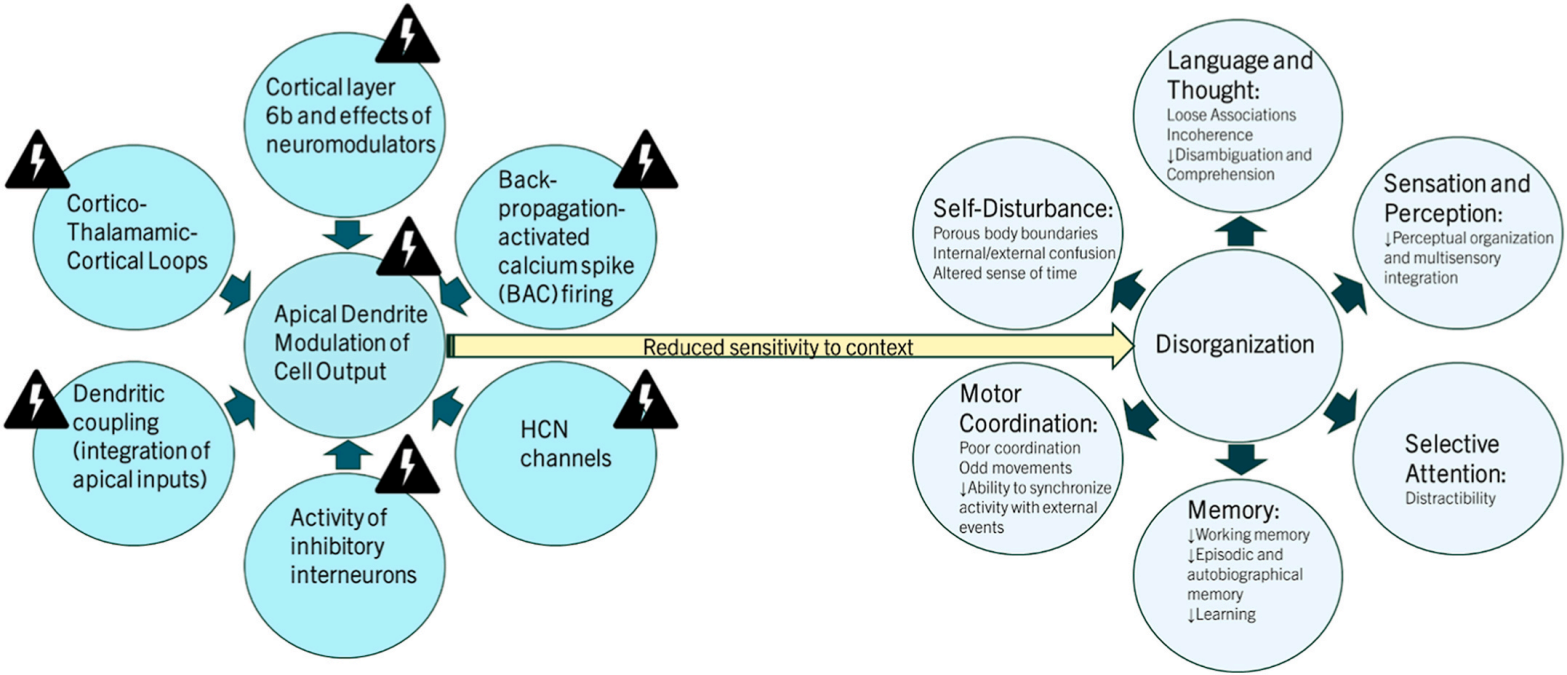
Multiple manifestations of disorganization hypothesized to occur in schizophrenia. Left - the neurobiological systems (discussed in Section 2) that appear to be disturbed in schizophrenia. Right: Resulting manifestations of disorganization.

Although disorganization in schizophrenia is typically discussed in terms of disturbances in thought and language, our view is that disorganization – with its basis in disturbances in the neurobiological systems reviewed above – manifests itself throughout the brain and therefore in multiple aspects of perception, cognition, subjective experience, and behavior. Below we review the domains for which this evidence is most suggestive (see also Fig. 6).

### 3.1 Perceptual organization

**Fig. 7:**
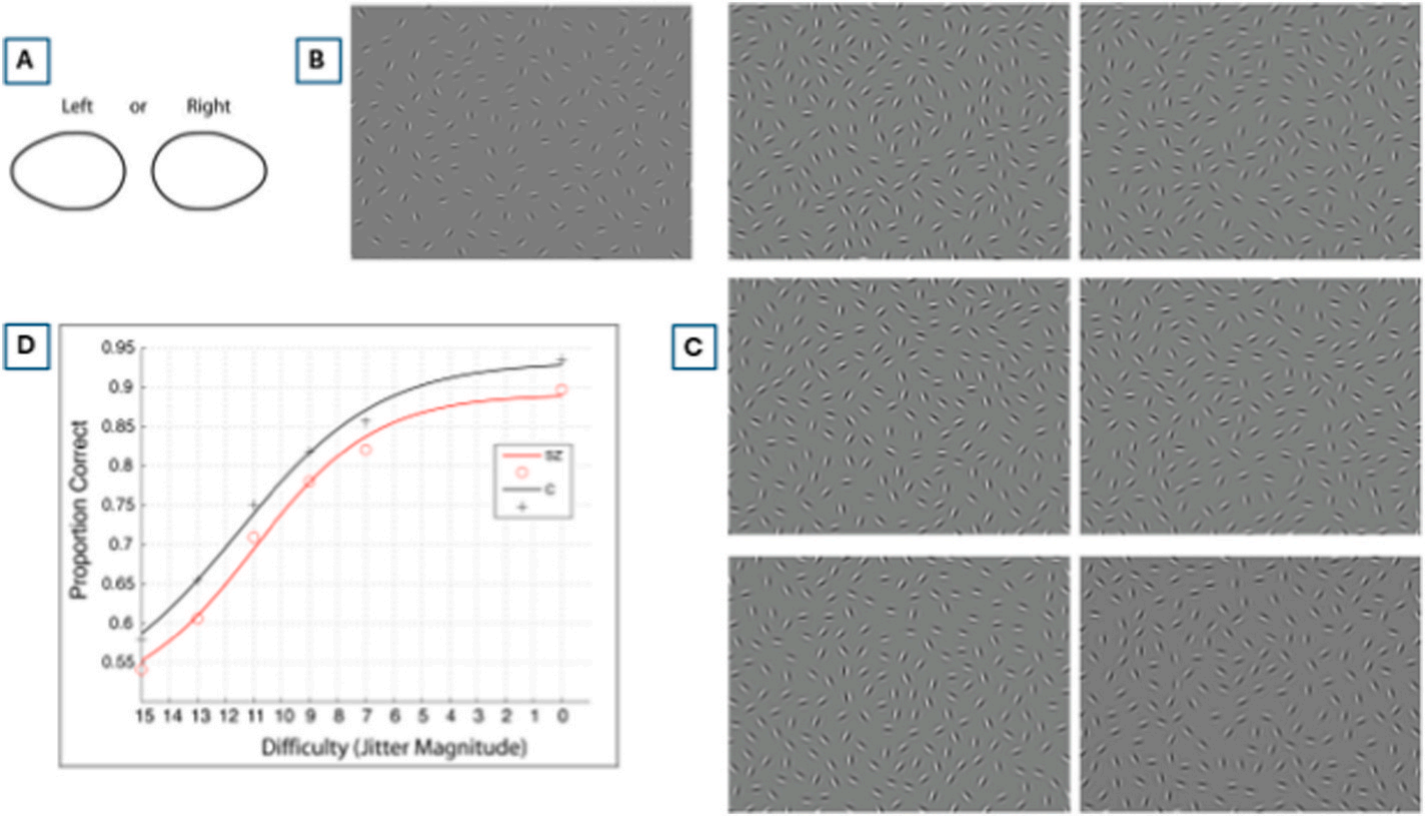
Stimuli from the contour integration task and representative data in schizophrenia. A) Examples of global shapes that appear on each trial. The subject's task is to determine whether the oval embedded in randomly oriented Gabor elements is pointing to the left or right. B) Example of a right-pointing stimulus from the condition where 0° orientational jitter has been applied to the contour elements. C) Examples of stimuli from conditions with orientational jitter applied to the contour elements. Left column: Top, ±7-8°; center, ±11-12°; bottom, ±15-16°. Right column: Top, ±19-20°; center ±23-24°; bottom ±27-28°. D) Psychometric functions from 124 healthy controls (C) and 99 people with schizophrenia (SZ). These data are typical of multiple published studies showing that even at 0^◦^of orientational jitter, patients display impairments, performing at a level that is similar to that of controls at 7° jitter. At 7° jitter, patients perform like controls at 9° jitter. It can be seen that people with SZ are responsive to the jitter manipulation but are less able to integrate individual elements into global shapes. Such performance in SZ is associated with normal activity in primary visual cortex (V1) but reduced activation in regions V2-V4 (Silverstein et al., 2009), and with reduced amplitude of the closure negativity (N_cl_) event related potential in the lateral occipital object recognition area at approximately 270 ms post-stimulus (Butler et al., 2013). It has been shown that this ERP is associated with successful perceptual organization.

The clinical literature on schizophrenia abounds with examples of perceptual organization impairment. Many examples concern vision [e.g. “I have to put things together in my head. If I look at my watch I see the watch, watchstrap, face, hands and so on, then I have got to put them together to get it into one piece” (p. 229) (see (Chapman, 1966) for this and other examples)]. However, this can occur with other senses (e.g., audition, gustation), as in the following example: “It's like eating a soup where you taste the individual ingredients: you taste the flavour of the soup itself only after reconstructing it” (Stanghellini, 2004) (p. 4). Many research studies also indicate that visual perceptual organization is impaired in schizophrenia (reviewed in (Silverstein, 2016; Uhlhaas and Silverstein, 2005; Silverstein and Keane, 2011)). This work indicates that impairments are greatest when fragmented stimuli need to be perceived as wholes, and especially when they appear within noise (see Fig. 1, Fig. 7 for examples), whereas perception of simple shapes with continuous contour is not affected. In general, the more that top-down input (based on memory, expectation, or strategy) is required for perceptual organization to occur, the more performance is impaired in people with schizophrenia relative to control subjects. The worsening of performance when top-down input is required has also been demonstrated in auditory perception (Silverstein et al., 1996). Performance on perceptual organization tasks is affected by both state and trait factors. It is lowest among patients with a history of impaired premorbid social and academic functioning (i.e., longstanding social and academic difficulties that predate the onset of psychosis, often beginning in childhood), poor prognosis, and impaired real-world functioning. Patients with a history of high functioning prior to psychosis onset generally display normal performance. This supports the idea that patients with perceptual organization dysfunction, and, we hypothesize, other forms of impaired cooperative context sensitivity, form a distinct subtype of patient, such as that classified under the term “process schizophrenia” (as opposed to “reactive schizophrenia”), and identifiable in cluster analyses (Farmer et al., 1983; Williams et al., 1993; Li et al., 2025). Notably, among patients with poor premorbid histories, perceptual organization breaks down further during states of acute psychosis. Multiple studies have noted the especially strong relationship between the degree of perceptual organization impairment and severity of disorganized symptoms, including conceptual disorganization (Silverstein, 2016; Phillips and Silverstein, 2003; Uhlhaas and Silverstein, 2005; Silverstein and Keane, 2011). Further supporting state contributions, improvement in perceptual organization occurs during treatment of acute psychotic episodes, where it covaries significantly with the magnitude of reduction in disorganized symptoms (Uhlhaas et al., 2005).

Zolnik et al. (2026) demonstrated that L6b drives sustained excitation of long-range neurons in the HoT and L5 that cross-connect CTC loops among distant cortical regions. This is thought to generate synchronization of gamma-band activity in different cortical regions and between thalamocortical loops (Zolnik et al., 2024; Fries, 2009), and to be the basis of amplification of related aspects of a mental representation (i.e., creation of figure that emerges against ground). Furthermore, since apical dendrites contribute to the tuning of cortical oscillations, sustained apical dendrite activity at specific frequencies may be required to promote binding of information (LaBerge and Kasevich, 2017). Schizophrenia patients have shown reductions in both synchrony and the gamma band activity that supports it during perceptual organization tasks (on which performance was abnormal), and these neural alterations have been related to the severity of disorganized symptoms (Grutzner et al., 2013; Uhlhaas et al., 2006). These observations are congruent with the notion that L6b plays a critical role in maintaining coherence and stability across thalamocortical loops, and, following from above, they suggest reduced activity at apical dendrites in schizophrenia. As noted above, binding and amplification of a subset of circuitry from among a total input field is thought to support perception as well as many other aspects of brain function. Hence, a widespread impairment in this process could result in increased neural noise, generation of weaker figures relative to ground (LaBerge and Kasevich, 2017), impaired performance on tests of perceptual organization such as that demonstrated in Fig. 7, and predictable relationships between reduced perceptual organization and other forms of disorganization in schizophrenia.

### 3.2 Multisensory integration

Multisensory integration provides a paradigmatic illustration of apical amplification. One example of this is that an abrupt onset of loud sound amplifies signaling in V1 but only for the cells that are selectively sensitive to the visual features currently being processed (Deneux et al., 2019; Ibrahim et al., 2016). A growing literature indicates that multisensory integration is impaired in schizophrenia, and that these impairments are related to symptoms such as hallucinations, reality distortion, and disorganization (Williams et al., 2010; Grohn et al., 2022; Poletti et al., 2026). Multisensory integration, especially involving tactile stimuli, is also critical for coherent self-experience (Tsakiris, 2008), including the experience that one has a single, whole body (Aspell et al., 2012). Impaired visuo-tactile integration has been implicated in various aspects of the ‘mirror sign,’ that is, disturbances in self-perception, in schizophrenia, such as self-other confusion, distorted perception of one's face, and feelings of unfamiliarity or estrangement when looking in the mirror (Poletti et al., 2026). A striking example involving impaired multisensory integration in schizophrenia can be seen in the following statement: “Consciousness gradually loses its coherence. One's centre gives way. The centre cannot hold … Sights, sounds, thoughts and feelings don't go together. No organizing principle takes successive moments in time and puts them together in a coherent way from which sense can be made” (Saks, 2007) (p. 13). The contributions of the integrative mechanisms we discussed in Section 2 to these phenomena have received little attention thus far, although there is some evidence for involvement of PV interneurons in multisensory integration (Choi and Lee, 2022).

### 3.3 Selective attention

As with perceptual organization and multisensory integration, selective attention is a form of cooperative context sensitivity that involves amplification of a subset of relevant stimuli and suppression of all others. That is, they involve the formation of a “coherence field” in which related representations are bound together, and segregated and prioritized over other information (Serences and Yantis, 2006). A clinical example of reduced selective attention in schizophrenia is as follows: “Nothing settles in my mind, not even for a second. It just comes in and then it's out. My mind goes away – too many things come into my head at once and I lose control” ((Chapman, 1966), p. 232). Impairments in both visual and auditory selective attention have been demonstrated in schizophrenia in laboratory tasks (Mathalon et al., 2004; Wood et al., 2006). Reduced selective attention has also been linked to psychotic symptoms (Malaspina et al., 2000), and poor attention loads highly on the Disorganization factor of the Positive and Negative Syndrome Scale [along with inappropriate (i.e., out of context) affect, conceptual disorganization, odd movements, and problems in abstract thinking] (Cuesta and Peralta, 1995; Lindenmayer et al., 1995). Selective attention has been theorized to depend on local activation by deep cortical L6b circuits, which are tightly regulated by a convergence of multiple neuromodulators. These L6b circuits in turn control the “volume” of each thalamocortical loop, making those that are activated more influential in the network. Loosening the neuromodulatory control on L6b would reduce the relative influence of the active loops, thus enabling “noise” to leak into the global state affecting attention and perception. Selective attention impairments in schizophrenia have been modeled as involving a weakening of the PV interneuron activity and gamma oscillatory power that normally implement segregation of streams of processing (John et al., 2018). This leads to “selective disinhibition,” a scenario analogous to the potential alterations underlying impairments in perceptual organization, memory, and language (discussed below) in schizophrenia.

### 3.4 Working memory

Working memory involves sustained amplification of novel combinations of elements and their segregation from noise, and thus it can be seen as an example of cooperative context sensitivity, analogous to selective attention and perceptual organization. Notably, it may be among the main functions of apical dendrite activity (Phillips, 2023). Working memory is thought to depend on the formation of reverberatory loops (as noted above, involving L6b activation and HoT-PFC loops) to sustain relevant activity after stimulus offset and to establish a set of features as the focus of attention. Feedback from the thalamus to the PFC is primarily to the apical dendrites in L1, and so those dendrites are a key link in recurrent PFC-thalamic loops. There is considerable laboratory evidence for impaired working memory in schizophrenia (Barch and Ceaser, 2012; Gold et al., 2019), both for auditory and for visual stimuli (Liu et al., 2025; Zhang et al., 2025). The clinical literature is also replete with examples, such as: “I can hear what they are saying all right, it's remembering what they have said in the next second that's difficult -- it just goes out of my mind” ((Chapman, 1966), p. 237). As is the case with perceptual organization and attention, poorer working memory is related to more severe disorganized symptoms in schizophrenia (Panov et al., 2023; Cameron et al., 2002; Perlstein et al., 2001; Barch et al., 2003). There is evidence that the working memory impairments that characterize this condition are associated with reduced gamma oscillatory power and, relatedly, cortical disinhibition involving reduced activity of PV interneurons (John et al., 2018; Starc et al., 2017; Lewis, 2012).

### 3.5 Episodic and autobiographical memory

Memory for events is also significantly impaired in schizophrenia (Danion et al., 2007; Kwok et al., 2021; Zhang et al., 2019). This includes difficulties in recalling contextual details of events, such as who was present, when an event occurred, and who performed which actions, even when the specifics of what occurred can be recalled (Rizzo et al., 1996; Danion et al., 1999). Much of this is thought to be due to an impaired capacity to link the separate aspects of events into cohesive and distinctive representations (Danion et al., 1999). Such binding deficits would serve as a rate-limiting factor in the dynamic amplification of the specific episodic memory traces relevant to a given event. It is not surprising, then, that schizophrenia is associated with an increased frequency of intrusions of involuntary autobiographical memories (i.e., those not due to conscious intention or effort to recall them) (Alle et al., 2020). Moreover, in the study by Allé et al. (Alle et al., 2020), the emergence of involuntary autobiographical memories into awareness was especially related in schizophrenia to internal (as opposed to external) triggers. This observation is consistent with the notion discussed throughout this paper that schizophrenia is characterized by an excessive influence of internal states on the generation of mental activity. It is notable that these authors found that intrusive involuntary memories were primarily related to everyday events with low emotional load, suggesting increased randomness in the landscape of memory representations, similar to what is observed in perception (see above) and language (see below) in schizophrenia. As noted previously, this may be related to reduced activity in SST interneurons, and a subsequent increase in interference among neural ensembles. Interestingly, in the study by Allé et al., both involuntary and voluntary memories reported by schizophrenia participants were rated as less clear and more poorly contextualized compared to those of controls. There is also evidence of reduced binding of self-representations into episodic memory traces in people with schizophrenia (Danion et al., 1999), which may leave patients with a sense of knowing that an event happened, but being unsure about their involvement in it. Finally, we wish to emphasize that while failures to contextualize information are related to the extent and accuracy of contextual information in memories of people with schizophrenia, there are other factors that influence memory consolidation in the disorder, including sleep disturbance (which also affects activity in thalamocortical loops) (Manoach and Stickgold, 2019).

### 3.6 Executive functioning

Executive functioning refers to a set of cognitive processes that support goal-directed behavior. Specific functions include switching of mental set, inhibiting the processing of irrelevant stimuli and responses, maintaining behavior consistent with specific strategies (including selective, sustained, and divided attention), and fluency. It is well-established that these processes, which are heavily subserved by the PFC (Friedman and Robbins, 2022), are impaired in schizophrenia (Orellana and Slachevsky, 2013). These deficits may be linked to failures in the specific cortical mechanisms discussed in Section 2. For example, a recent mouse study (de Las Casas et al., 2026) demonstrated that experimentally-induced inhibition of activity at apical dendrites of L5 neurons disturbed the adaptive relearning of a complex rule during a rule-switching task, whereas previously-learned behaviors were unaffected. This finding indicates that integration of apical inputs is important for flexibly adapting to new contexts. Two-photon calcium imaging demonstrated that calcium spiking was suppressed in dendritic shafts but not in individual spines, indicating that while inputs were being processed at individual spines, they were not being integrated. Perhaps most remarkably, synapses at L1 apical dendrites of L5 cells displayed functional clustering, with subsets of synapses becoming co-active based on which rule was operational (task context). Depending on the behavior required, these excitatory inputs were implemented by thalamocortical or cortico-cortical loops. In short, adapting behavior to new task contexts involves integration of apical input at the level of the dendritic shaft, and grouping of apical inputs based on long-range input from other brain regions. These findings suggest that executive functioning deficits in schizophrenia may be mediated by impairments in the processes that implement apical integration. In addition, alterations in the PFC in schizophrenia, such as dendritic spine loss, reduced activity at PV interneurons, and relatedly, decreased inhibition, are thought to disrupt both microcircuits within the PFC and coordination between the PFC and other brain regions, thereby impairing perception, cognition, and behavior [see (Cheng et al., 2025) for review]. In general, however, much remains to be learned regarding the role of the circuity we described above in executive functioning impairments in schizophrenia.

### 3.7 Learning

Synaptic plasticity is one of the biological mechanisms through which learning can occur, and this involves the context-sensitivity of apical dendrites (Benezra et al., 2024). Learning is also dependent on several of the cognitive processes discussed above, including executive functions, which, as noted, are supported by apical amplification. Multiple forms of impaired learning have been demonstrated in schizophrenia, including perceptual (Silverstein and Keane, 2009), associative (Diwadkar et al., 2008), implicit and explicit (Horan et al., 2008), and reinforcement-based (Barch et al., 2017). Some of this work, along with psychiatric rehabilitation research, indicates that learning in schizophrenia tends to be slower and to be less facilitated by top-down cues compared to learning in healthy controls. Thus, for learning goals to be met by participants with schizophrenia, training typically requires more frequent repetition, more structure to the presentation of stimuli, and more explicit linking of reinforcers to performance (Silverstein and Keane, 2009; Saperstein and Kurtz, 2013; Silverstein, 2010; Silverstein and Wilkniss, 2004; Silverstein et al., 2006). Schizophrenia-related impairments in synaptic plasticity and learning are thought to arise due to multiple alterations, including dendritic and synaptic abnormalities related to DISC1 mutations (Obi-Nagata et al., 2019)., as was the case with HCN channels (Paspalas et al., 2013; Roberts, 2007).

There are two aspects of learning that are often ignored in discussions of schizophrenia but that are relevant to understanding the nature of learning problems in, and design of psychological interventions for, people with this diagnosis. One component involves affect. The brain integrates emotional content into memory representations, and this helps establish positive and negative valences associated with events, in addition to enriching self-understanding and life narratives (Cocquyt et al., 2025, 2026). A reduced ability to integrate affective content into memory representations would both impair learning and lead to self-representations that are less grounded in, and more alienated from, bodily experience, including emotion-related arousal. Although we are not aware of studies directly testing this hypothesis, we view it as consistent with findings pointing to a marked impairment in integrating contextual information into episodic memory representations (Talamini et al., 2010), and to reduced coherence in life narratives, including less causal-motivational coherence (Alle et al., 2015), in schizophrenia. We propose that reduced integration of affect into ongoing memory formation is related to several forms of self-disturbance commonly observed in schizophrenia, including nihilistic delusions, such as believing that one does not exist or that one is dead. It may also be related to the common delusion that one's body has been turned into a machine. Reduced integration of affect into memory traces may be a setting condition for inappropriate affect and for the splitting of affect and ideation in schizophrenia (Moskowitz and Heim, 2011). On the other hand, it has been suggested that autobiographical memories in schizophrenia may be organized more prominently by emotional content, to compensate for impairments in using sensory and cognitive information to structure memories (Morise et al., 2011). It is thought that such alterations may play a role in the increased rate of false autobiographical memories in people with schizophrenia (Pernot-Marino et al., 2010) and in delusion formation.

### 3.8 Fragmentation in language and thought

What follows is an extended discussion of language disturbance in schizophrenia. We emphasize this material for four reasons. First, language disturbance is prominent in schizophrenia (Ehlen et al., 2023; Jo et al., 2023). Second, as noted by Phillips (2023), language and thought are highly dependent on the use of context to disambiguate the meanings of items within dynamically created syntactic and semantic structures. Third, the eminent researcher and theoretician of schizophrenia, Tim Crow, hypothesized that schizophrenia is the price that *Homo sapiens* pays for language (Crow, 1997). Specifically, language allows for expanded representations of relationships between things and categories of things, and for reflections on the self. As Crow described, a consequence of evolving such capabilities is that these processes can go awry, leading to, for example, the excessive flexibility in the formation of concepts, use of language, and self-understanding that is characteristic of many people with schizophrenia. Fourth, we view language disruption in schizophrenia as relevant to the nature of consciousness in this condition, which is the focus of section 4, below.

In schizophrenia, disturbances in expressive language, such as loosening of associations and fragmentation (and sometimes incoherence) in speech, along with difficulties in language comprehension, are commonly observed. These language-related impairments are independent of negative thought disorder (e.g., poverty of speech) and overall symptom severity, suggesting that they are manifestations of a separable core impairment (Sharpe et al., 2026). It has been shown that impaired language comprehension (Hoffman and McGlashan, 1993; Cohen et al., 1999; Condray et al., 1999; Kuperberg et al., 1998, 2000) and production (Barch and Berenbaum, 1996; Brown and Kuperberg, 2015; Spitzer et al., 1994) in schizophrenia are related to reduced context sensitivity. For example, research has suggested that the fragmentation in thinking and loose associations in speech that characterize the condition are driven by a weakening of the normal predictive constraints that words or ideas have on the activation of subsequent words and ideas (Sharpe et al., 2026; Spitzer et al., 1994; Matsumoto et al., 2026). Earlier work described a reduction in the use of linguistic context during a verbal recall task in thought-disordered patients (Maher et al., 1980).

Findings from multiple studies suggest that disorganized speech and impaired perceptual organization in schizophrenia arise due to shared computational failures (reviewed in (Phillips and Silverstein, 2003; Uhlhaas and Silverstein, 2005; Silverstein and Keane, 2011). These data are consistent with theories positing structural similarities between linguistic and visual representations (Arnheim, 1969; Logan, 1999; Glezer, 1995; Fuster, 2000) and shared neural implementation in percepts and semantic representations (Mesulam, 1990; Pulvermuller, 2018). For example, it has been proposed that feature representations for visual patterns and objects use propositional representations similar to those in language (Logan, 1999; Chechile et al., 1996), and that language and perception evolved from the same processing algorithms for extracting regularities from the environment (Cavanagh, 2021), a position with obvious overlap with our view of the function of apical dendrites.

As Phillips (2023) noted, the dependence of semantic memory and language organization on apical dendrites has not been demonstrated to the extent that it has for other domains such as perceptual binding, selective attention, and disambiguation. Data on language processing in schizophrenia can inform this issue, as it demonstrates that organization of language in this condition is characterized by reduced contextual modulation and increased randomness, as is the case in perception and memory, where support for involvement of apical integration is stronger. For example, Hayashi et al. (2024) demonstrated that compared to controls, word concepts in people with schizophrenia were represented with a reduced set of unique associations that depended on context or abstract/figurative meanings. Moreover, increased randomness in linkages between word meanings was related to increased severity of delusions. Similar findings were obtained by Camon et al. (2025), who examined structural speech graphs of written and oral dream reports from 115 individuals with acute psychosis and 78 healthy controls. The key result was that psychotic patients exhibited significantly lower connectivity values than controls, with their speech graphs more frequently resembling random word sequences. Other findings in support of weaker connections between words that are conceptually related come from a study by Lundin et al. (2020), which found that in a verbal fluency test, schizophrenia patients showed longer switching times between words within the same category, but similar switching times to controls when switching between categories. This was especially true in thought-disordered patients. Kreher et al. (2009), using an event-related paradigm, reported increased priming to indirectly related primes in thought-disordered patients but not in non-thought-disordered patients or controls, reminiscent of the randomness in semantic networks observed by Hayashi et al. (2024). Finally, Rezaii et al. (2019) used a machine learning approach to predict development of psychosis among people at clinical high risk for a psychotic disorder using semantic density and latent content analysis of speech samples. Their main finding was that low semantic density predicted conversion to psychosis. This index can be considered a measure of the level of organization in speech because it depends on the way the words are grouped together into sentences and not simply on the set of words used in the sentences. A conclusion from this study was that semantic density is sensitive to the way words are grouped, and thus with the processes involved in combining them into sentences.

Based on the data reviewed in the preceding paragraph, we propose that fragmented thinking and speech in schizophrenia reflects reduced context-based connectivity in storage and retrieval of linguistic concepts, in addition to reduced disambiguation in language production and comprehension processes. This is compounded by a faster and wider spread of activation in semantic networks in schizophrenia, which is most pronounced in patients with observable thought disorder (Kreher et al., 2008). While there is no direct evidence at this time that this reflects reduced activity at apical dendrites (and thought disorder has the distinct disadvantage that it cannot be studied in animals), it is notable that these symptoms are at the core of the disorganization syndrome, which includes symptoms such as cognitive impairments and inappropriate affect that can also be seen as reflecting a weakening of cooperative context sensitivity (as described above), as can reduced perceptual organization. Moreover, reduced sensitivity to global context in language production in schizophrenia is associated with reduced cortical glutamate levels, even after controlling for other clinical and language variables, (Wang et al., 2025), and thought disorder has been linked to NMDA-mediated recurrent inhibition (interneuron) abnormalities that lead to spreading activation in semantic networks (Nestor et al., 2001), both of which are consistent with our hypotheses. As we have argued is the case for perceptual organization, selective attention, and memory, it is possible that aspects of formal thought disorder in schizophrenia (e.g., loose associations, fragmented thinking) arise from weakened effects of context on processing, reduced inhibition of normally irrelevant (in the current context) representations, and poorly segregated streams of processing. Kuperberg et al. (2010) also concluded that the structure and function of semantic memory, and abnormalities in combining and integrating words together to build up sentences, are not independent, and that together, impairments in these systems in schizophrenia place limits on the representation of higher-order meanings.

### 3.9 Other cognitive processes

In addition to the functions reviewed above, reduced context sensitivity has been observed in a range of other cognitive processes in schizophrenia. These include reduced effects of irrelevant letters flanking a target letter during a choice reaction-time task (Jones et al., 1991), reduced effects of prior context on reaction time (Nuechterlein, 1977), reduced pre-pulse inhibition (Adler et al., 1998), reduced effects of context in weight perception and related paradigms (reviewed in (Magaro, 1980)), reduced mismatch negativity amplitudes (generated when a stimulus violates a repeating stimulus pattern) (Catts et al., 1995; Mazer et al., 2024), reduced coordination in motor behavior (Carr and Wale, 1986; Varlet et al., 2012), disturbances in the experience of the temporal continuity of time (Vogeley and Kupke, 2007; Vogel et al., 2019; Fuchs, 2007; Foerster et al., 2024), and a reduced ability to synchronize actions with external events (Wilquin et al., 2018). Several of these impairments, as well as those discussed above, are considered potential endophenotypes and/or biomarkers of core alterations in schizophrenia (Silverstein and Keane, 2011; Greenwood et al., 2019a, 2019b; Sierakowska et al., 2025). Additionally, in the case of mismatch negativity and sensory gating disturbances in schizophrenia, evidence indicates the involvement of reduced activity of PV and SST interneurons (Lakatos et al., 2020; Ross and Hamm, 2020), and in the former case, reduced activity at thalamic projections to pyramidal cell dendrites in L1 (Lakatos et al., 2020). Other work has proposed that the reduced inhibitory effects involved in selective attention impairments in schizophrenia are also related to problems in smooth pursuit eye movements/tracking (John et al., 2018). The latter is an impairment in schizophrenia (Meyhoefer et al., 2024) that has not been the focus of prior work on impaired contextual modulation in the disorder.

### 3.10 Functional consequences of impairments in cooperative context sensitivity in schizophrenia

The impairments discussed in subsections 3.1 - 3.9 all involve consequences of a reduced ability to amplify representations of relationships between concretely (e.g., as in perception) or conceptually (e.g., as in language) related aspects of mental activity, be they internally or externally driven. As we proposed in an earlier paper (Silverstein et al., 2017), this reduced connectedness can be expected to increase the uncertainty (entropy, in the information theory sense) associated with any single mental representation (e.g., visual feature, object, scene, word, etc.). This would occur because the number of plausible interpretations of a stimulus or scene would increase, as would the number of likely associations that could be generated, thereby loosening constraints on the perceived nature of, and the response requirements associated with, the stimulus or scene. We hypothesized that this increase in processing demands could lead to slowed processing, increased distractibility, an increase in the likelihood that an irrelevant stimulus or association would be paired with relevant information, and the generation of behavior that appears unrelated to the current context (i.e., bizarre or disorganized behavior). This is supported by the much-replicated association between disorganization and cognitive impairment in schizophrenia (Lindenmayer et al., 1995; O'Leary et al., 2000; Vignapiano et al., 2019).

### 3.11 Psychotic symptoms

The underlying mechanisms of psychotic symptoms such as hallucinations and delusions in schizophrenia remain largely unknown. However, accumulating evidence suggests that psychotic symptoms arise due to weak top-down predictive signaling and a related overweighting of sensory evidence during perception. It is thought that this set of alterations allows unreliable sensory input to drive inappropriate updates to priors, resulting in erroneous inferences (Sterzer et al., 2018). Multiple findings provide support for this framework (Keane et al., 2013; Petrovic and Sterzer, 2023) However, there is also evidence suggesting the opposite – that is, that psychosis is characterized by excessive top-down signaling (or an increased precision of priors), which leads to a greater weighting of expectations and past experience in determining perception and belief (Aleman et al., 2003; Teufel et al., 2015). For example, influential findings have linked hallucinations with a perceptual bias towards expectations (Cassidy et al., 2018; Corlett et al., 2019; Powers et al., 2017). In efforts to reconcile what seems to be incompatible findings, recent accounts have emphasized the hierarchical aspects of predictive processing, proposing, based on multiple findings, that there may be a weakened influence of priors at low levels of sensory processing, and, as a result of an overreliance on noisy sensory evidence at these levels, a compensatory strengthened influence of priors at higher processing levels (Petrovic and Sterzer, 2023; Corlett et al., 2019; Schmack et al., 2017). It is possible that these seemingly conflicting findings can be reconciled by testing whether the observed weak experimenter-generated top-down effects are overridden by excessively strong top-down contributions stemming from learning history and related longstanding cognitive schemas and emotional responses, which are not typically assessed in this research. It is also possible that in some cases, weaker experimenter-induced top-down effects may arise due to working memory deficits or other impairments that may be more pronounced in participants with more severe psychotic symptoms.

The apical dendrite and neuromodulation mechanisms discussed above provide an alternative view, or potentially a complementary elaboration, of positive symptom genesis. Recent studies of the function of apical dendrites indicate that there can be transient periods of apical overdrive (associated with stress, intense emotion, hyper-arousal, and threats to self) in which contextual information can cause action potentials (i.e., serve as feedforward input to the next level of processing, as opposed to having only contextual and modifying effects on the strength of output signals, as is typically the case). This would essentially represent an overdriven form of apical activity that, under less extreme circumstances, generates experiences such as perceptual filling-in, imagination, or dreams. Apical drive implies that internal and emotional context (e.g., shame, fear, rage) provide both contextual and feedforward activity. The resulting distortions in perception and the evaluation of events are then experienced as real because they are strongly related to the predominant internal context. Phillips (2023) proposed that positive symptoms may thus arise from transient periods of apical overdrive that occur within the context of a trait-like apical underactivity (and thus reduced context sensitivity).

## 4 The nature of disturbed consciousness in the schizophrenias

Although schizophrenia is characterized by a range of perceptual and cognitive alterations, it is important to note that this condition is not one of normal consciousness accompanied by cognitive limitations and reduced perceptual sensitivity. As noted by Garrett (2019), “Psychosis transforms the contours of consciousness. It creates a new mental space that has different rules from the more familiar forms of consciousness” (p. 47). In particular, psychosis disturbs the mechanisms that generate an embodied and agentic sense of self and that allow for a clear distinction between what arises from one's own thoughts and body versus from external stimulation. We argue below that the mechanisms involved in these fundamental disturbances may involve those that are the focus of apical integration theory. Subsequent efforts by the person with these impairments to create meaning out of their experience may lead to many of the psychotic symptoms reported by patients, including beliefs about thought broadcasting and insertion, changes in identity of self or others, and the reality of otherwise non-realistic facts, events, and states of the world. What is thought to enable the unusualness of the resulting beliefs is that meanings can be generated not on the basis of logical thought, but rather, on the basis of weakened effects of language on thought and strengthened effects of concrete, sensorimotor representations, emotional states, and instinctual drives (e.g., aggression, sex, security) (Garrett, 2019); see also section 4.1 below).

One view of consciousness proposes that it emerges via the selective apical amplification of currently relevant signals (LaBerge, 2006; Laberge and Kasevich, 2007) and the broadcasting of these signals to multiple brain regions (Marvan et al., 2021; Dehaene and Changeux, 2011). This view is consistent with work suggesting that the phenomenology of conscious experience – that is, the nature of experience - is more dependent on the structure of neuronal connectivity than it is on the nature of the content being processed (Malach, 2021; Storm et al., 2024; Song, 2021). If this is so, then in schizophrenia, in which connectivity is weakened, conscious representations and motoric actions may be experienced as less rich and potentially as less “personal,” and instead as alien to the self, imposed upon (or removed from) the self (as in Schneiderian First Rank Symptoms (Schneider, 1959)), or even as occurring outside of the self, as in hallucinations. Related to our view that cooperative context sensitivity is disturbed throughout the cortex in schizophrenia, Melges and Freeman (1977) found that severity of first-rank symptoms in schizophrenia, which can be viewed as reductions in the ability to distinguish between inner versus outer experience, correlated significantly with a disturbance in temporal organization in patients. In a broader sense, because selective amplification is dynamically adaptive, given the constantly shifting array of contextual inputs from diverse sources, including the PFC, reduced contextual input in schizophrenia could be a setting condition for alternate contexts to dominate, and for alternate meanings to emerge.

The contribution of apical input to the later component of neural firing in sensory areas and to contextual modulation and awareness of sensory stimuli (Lamme et al., 2000; Manita et al., 2017; Lee et al., 1998), and the roles of dendritic calcium spiking and NMDA receptors for perceptual awareness, motor coordination, attention, and self-awareness (Manita et al., 2017), suggest that the mechanisms discussed in Section 2 are relevant to typical conscious experience and to disordered consciousness as observed in schizophrenia. Furthermore, each of the components of the system involved in apical amplification may have a critical role in consciousness (e.g., cortico-cortical and cortico-thalamic loops, along with modulation of, and maintenance of activity in, these loops by neuromodulators that regulate arousal and wakefulness). Reverberant activity can also be maintained in these loops via strong direct excitatory connections from the thalamic nuclei to neurons in layers 2 and 3, which then activate cells in layer 5, which activate thalamic nuclei (Constantinople and Bruno, 2013).

**Fig. 8:**
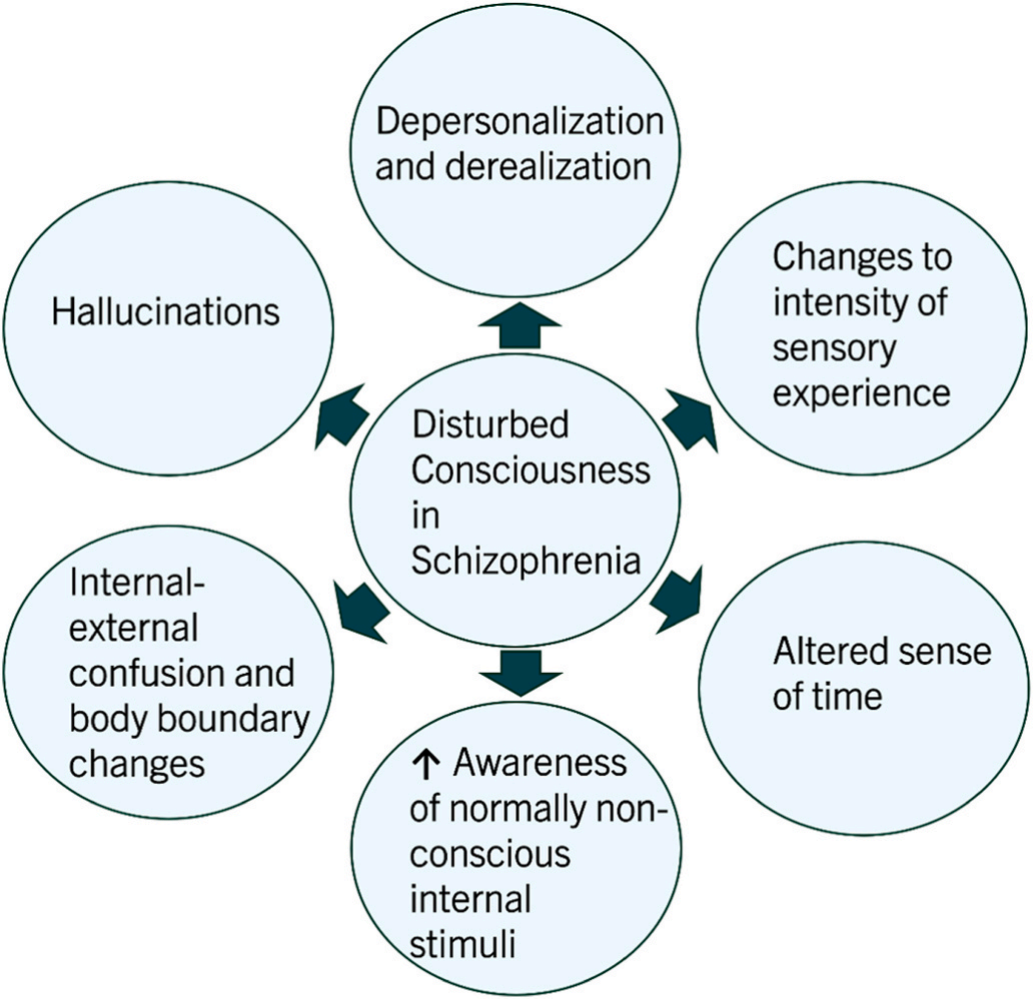
Key manifestations of consciousness disturbance in schizophrenia.

We mentioned in the Introduction that an early view of the core feature of schizophrenia was that of a ‘lowering of the level of consciousness’ (*abaissement du niveau mentale*). This fits with a recent theory (Berkovitch et al., 2017) positing that schizophrenia is characterized by an “elevated consciousness threshold,” or a condition where recurrent processing is deficient and so many stimuli that cross the threshold of consciousness in other people are processed but remain at the preconscious level. This is a rate-limiting factor for emergence of figure from ground, a clear focus of attention, and subsequent inhibition of unrelated mental activity. In Berkovitch (Berkovitch et al., 2017), data were interpreted according to the Global Neuronal Workspace (GNW) theory of consciousness (Dehaene et al., 1998; Mashour et al., 2020), which involves recurrent networks, top-down amplification of signals, and ‘ignition’ wherein representations that cross a certain threshold of activation are ‘broadcast’ to multiple regions of the cortex via a network that includes the PFC. In addition to positing that an elevated threshold of consciousness is caused by inadequate amplification of sensory stimulation, there is also recognition in this model that sensory information may be too weak to entrain amplification processes. Evidence from paradigms such as electroretinography (Demmin et al., 2018; Silverstein et al., 2020), visual evoked potentials (Silverstein and Butler, 2026) and others (Javitt and Freedman, 2015) indicates a reduction in the strength of sensory signals in schizophrenia, and this may play a role in contributing to an elevated threshold of consciousness. Reduced drive on CTC loops, as discussed above, may also be a biological basis of reduced signal strength and reductions in consciousness in schizophrenia. See Fig. 8 for a graphical depiction of the proposed manifestations of consciousness disturbance in schizophrenia.

While the above situation is thought to characterize many people with chronic schizophrenia, people in the earliest stages of psychotic illness (i.e., with an emerging and as yet untreated first episode of psychosis) may, in some cases, be characterized by excessive sensory activity [due, e.g., to active neuroinflammation (De Filippo and Schmitz, 2024), thickening of retinal neural layers (Demirlek et al., 2026), phase-related increases in glutamate levels (Rivera-Chavez et al., 2026), etc.], which may account for findings such as increased contrast sensitivity and hypersensitivity in face perception in the early phases of the condition (Silverstein, 2016; Silverstein et al., 2021; Tran et al., 2025). There may also be later periods of excessive sensory activity during relapses that can be superimposed upon a progressive neurodegenerative process, consistent with the apical drive hypothesis of Phillips (2023) noted above. This raises the question of what the combined effects would be of transient excessively strong sensory activity, or neural activity more generally, occurring against a background of weak top-down amplification. We suggest that this would include an enhancement of ‘ground’ processing, or a reduced signal-to-noise ratio. In this scenario, novel ‘figures’ – including from a hyper-awareness of one's internal physical or mental states (i.e., an extreme form of self-consciousness, referred to as hyper-reflexivity (Sass et al., 2018)) -- could be formed and stabilized, leading to the initial emergence of novel meanings for situations, false beliefs, hallucinatory experiences, and other examples of novel networks whose activity is overly independent of the state of the world (i.e., of other network activity). Although the goal of discovering relationships is important for both learning and prediction, the sustained signaling of relationships that are weak or overly personalized is typically maladaptive. If the threshold for signaling a relationship is too low (perhaps as compensation for weak sensory signaling), the normally dominant responses to stimuli will be given less prominence relative to other potential response options, and normally weak responses will become more likely to enter consciousness, function as context, and guide behavior. An example of this relative equalization of all possible contexts, as it occurs in schizophrenia, was reported by a patient of Matussek (1952), quoted above. The idea that the emerging psychotic state is one in which novel associations are likely to be formed, and that these are maintained later by both their linkages with personal and emotional issues, and by their disconnection from other, more reality-reflecting, neural circuits, is consistent with data demonstrating hyperactivation of default mode network regions and hyperconnectivity of the default network in schizophrenia patients and first-degree relatives (Whitfield-Gabrieli et al., 2009). These disturbances were hypothesized to blur the distinction between internally and externally generated stimuli, and they were related to positive and disorganized features in both study samples. Another study demonstrated hyperconnectivity in cerebello-thalamo-cortical circuitry in people at clinical high risk for psychosis that was related to severity of disorganized (but not other) symptoms and predictive of later development of a psychotic disorder (Cao et al., 2018). A third study showed hyperconnectivity within the PFC that correlated with the extent of psychotic symptom emergence after drug use, and that was similar to findings in people at high risk for psychosis and people with a recent first psychotic episode, but not in patients with chronic schizophrenia (Anticevic et al., 2015), suggesting a key role for hyper-connectivity in the initial development of impaired reality testing. Among chronic schizophrenia patients, disorganization has been linked to reduced connectivity within frontal regions (Yoon et al., 2008), suggesting that the abnormal neural connections formed during the early stages of psychosis may be maintained, at least in part, by their later disconnection from other brain activity.

### 4.1 Schizophrenia, consciousness, and language

Phillips (2023) suggested that there may be a deep analogy between the essential nature of amplification and the semantics of language. Amplification strengthens the transmission of information by framing it within the characteristics and requirements of the current context, and word representations allow for the thing represented to be conceptualized within a broader context as a result of their relationships with dissimilar things that share abstract relationships (e.g., bicycle-car-boat-airplane-camel). Phillips hypothesized that language capabilities may have arisen via a transformation of amplifiers into generators of information. We see this as relevant to schizophrenia, based on Freud's (Freud, 1915) distinction between ‘the idea of the word’ [i.e., the linguistic code representation (word)] and ‘the idea of the thing’ (i.e., the concrete sensorimotor impressions and memories of the thing itself). This distinction goes back to ideas first put forth by Freud in his 1891 neurological work, *On Aphasia* (Freud, 1953), and is consistent with both Wernicke's (Wernicke, 1874) earlier distinction between words and word concepts and modern research on different systems for naming and recognition, and for distinctions between verbal language systems and sensory-motor representations of objects (Damasio et al., 2004; Amit et al., 2017; Kemmerer, 2026). In Freud's view, conscious thought was seen to involve hyper-excitation (cathexis) of the sensorimotor representation by linking it with a linguistic representation. In contrast, unconscious representations are thought to involve only sensorimotor representations without linguistic context or constraint. It is possible that schizophrenia, as a consequence of generally reduced apical amplification, involves weaker-than-normal connection strengths between word representations and their normally associated thing representations, allowing for sensorimotor (“thing”) representations to more flexibly become associated with other sensorimotor representations and with novel word representations. This would lead to novel conceptual and word associations, idiosyncratic assignments of meaning, and to the interpersonal use of language in odd ways. As noted in section 4.0, the relative increased influence of normally non-conscious and non-linguistic (i.e., sensorimotor) representations on consciousness is a possible factor in the shaping of psychotic beliefs out of altered perceptual and self-experience (Garrett, 2019).

A simple example of this can be seen in the response of a patient of the first author to the question: “How are a fly and a tree alike?“, to which the response was, “They both have leaves.” A related example, from Cutting and Dunne's influential paper on subjective experience in schizophrenia (Cutting and Dunne, 1989), is from a patient who stated: “I was functioning on another level, one more in pictures, then another one – the abnormal one was the picture one” (p.230). A more complex example was described by Freud (Freud, 1915). This patient stated that her “eyes were not right, they were twisted.” She went on to note the following about a man she knew, whom she blamed for this condition: “She could not understand him at all, he looked different every time; he was a shammer, an eye-twister; he had twisted her eyes, now they were not her eyes anymore; now she saw the world with different eyes.” In this case, the associations to an internal state (e.g., feeling rejected, angry, not oneself, unable to understand her ex-lover who could be dishonest and disingenuous) were bound together especially strongly with a normally distant association, in this case, the eyes (i.e., cannot understand → cannot see → eye problem), leading to the concretization of this association into a belief in an actual physical change (twisted eyes). About this process, Freud (Freud, 1915) noted that “In schizophrenia, words are subject to the same process as that which makes dream-images out of dream-thoughts, the one we have called the primary mental process. They undergo condensation, and by means of displacement they transfer their cathexes to one another without remainder; the process may extend so far that a single word, which on account of its manifold relations is especially suitable, may come to represent a whole train of thought. The works of Bleuler, Jung, and their pupils have yielded abundant material precisely in support of this very proposition (p.186).” Translated into the language of modern neuroscience, what this example demonstrates is that the normal contextual field that would be associated with an experience such as rejection is weakened, allowing for distant and atypical (but not random) associations to become strengthened and to emerge into consciousness. Comparing this to Mattusek's patient with emerging psychosis noted above, who said that his “ability to see connections had been multiplied many times over,” the patient Freud described is characterized by a more severe degree of loosening of associative links, more vulnerability to forming and maintaining new (distant) associations, a loss of awareness that the latter is occurring, and therefore the construction of a revised (psychotic) model of the world in which the new associations are taken for granted as a new reality.

Another example of the lack of normal contextual constraints allowing for novel linkages between words and feelings was provided by Jung (Jung, 1960). His patient, when trying to express that she had been the finest dressmaker, stated “I am double polytechnic irreplaceable” (p. 114). In this case, a novel combination of words was linked to a feeling of (compensatory) superiority, as was seen in the following sequence of associations produced by the patient: “That is the highest → all highest → the highest of dressmaking → the highest achievement → the highest intelligence → the highest achievement in culinary art → the highest achievement in all spheres → the double polytechnic is irreplaceable …” As in the example in the above paragraph, this demonstrates an associative chain based on an internal state (i.e., a compensatory feeling of superiority), and a loss of insight that the associations reflect this internal state and not external reality. In addition, the term ‘double polytechnic irreplaceable’ integrates the concepts of education/intelligence (polytechnic), importance (irreplaceable), and extraordinariness (double) into a novel word triad that has personal meaning in the context of the patient's life, but that is incomprehensible to others.

While we have focused on thought and language in this section, it is important to note that the mechanisms that are the focus of this paper also apply to self-disturbance. For example, when L5-ET neurons send motor signals to the brainstem and spinal cord, there is a second signal to HoT that is relayed back to L1 in the cortex, but also to other input layers. This second signal can be considered an efference copy that informs much of the cortex that an internal signal to move was just sent (Guillery and Sherman, 2011). If L6b activation of thalamo-cortical loops is deficient in schizophrenia, as we hypothesize, then the efference copy signal from HoT to cortex would be degraded and the subsequent movement would be experienced as not caused by the self. Similar examples of altered efference copy signals may be operative in schizophrenia regarding sensory stimuli as well, leading to misperception and subsequent elaboration (driven by emotion) of internal speech, visual imagery, and thought as external stimuli (e.g., auditory and visual hallucinations, first-rank symptoms such as thought insertion). This view is supported by data suggesting a generalized problem generating efference copy signals in schizophrenia (Pynn and DeSouza, 2013). This scenario could contribute to confusion between self- and non-self and to the more porous and flexible body boundaries observed in schizophrenia and psychosis-prone individuals, as well as to delusional passivity experiences (Benson and Park, 2019; Giersch et al., 2025).

In summary, consciousness in schizophrenia can be said to be altered in multiple respects. Reduced cooperative context sensitivity could cause reduced organization in perception and thought, allowing for intrusion of mental content that would normally not reach the level of consciousness, novel combinations of ideas and images, and novel and sometimes creative meanings to be assigned to experiences. This tendency may be especially pronounced in the period before, and during, a first psychotic episode. However, once the new meanings take on a taken-for-granted quality and in a sense become part of the new contextual field for everyday understanding, they become more resistant to dissolution and begin to contribute increasingly to the ongoing organization of, and creation of meanings for, new experiences, similar to how a complex or an attractor state expands and stabilizes. Subsequently, delusional ideas are maintained because they make more sense to the patients than do alternative explanations offered by mental health professionals or other people (Freeman et al., 2004), especially regarding patients' internal anomalous experiences (e.g., perceptual distortions, sense of lack of control over one's thoughts and movements, etc.). Moreover, conviction serves a psychological benefit: Having doubt about delusions without having an alternate explanation is associated with lower self-esteem (Freeman et al., 2004), and many delusional beliefs serve a compensatory function in relation to low self-esteem (e.g., “I am double-polytechnic irreplaceable”), in the sense that it is better to believe one is a highly educated and essential-to-society person placed against their will in a psychiatric hospital due to some conspiracy than to see oneself as a person with schizophrenia who cannot function outside of a hospital (see (Davidson, 2003)). Finally, a saying traditionally attributed to Kraepelin is that schizophrenia is like ‘an orchestra without a conductor.’ To this we add that it can also be usefully seen as a set of orchestra musicians, each of whom is able to process only what the musicians closest to them are playing (analogous to failures in long-range contextual modulation), while at the same time operating with an increased sensitivity to internal cues (e.g., the feelings of the back of their legs against the chair, intrusive thoughts that break the frame of focusing on the music) and to aspects of context that would normally not become conscious (e.g., breathing sounds of nearby musicians, coughing in the audience, brightness of an overhead light, etc.). While musicians in this situation would be able to play music, it would become increasingly comprehensible only to themselves and increasingly unrelated to what was happening around them.

## 5 Discussion

The main goal of this paper was to demonstrate that recent discoveries involving two-point neurons, the context sensitivity capabilities they provide, and the neural circuitry and modulatory systems in which they are embedded, may be relevant for understanding disorganized, and possibly other, symptoms of schizophrenia. These discoveries, by helping to better characterize the disorganization syndrome and changes in its expression from the prodromal to chronic stages of the disorder, could help better characterize heterogeneity within ‘the group of schizophrenias,’ (Bleuler, 1950) and ultimately inform the development of more effective treatments for these symptoms. It was also our explicit intention to demonstrate that the functions of two-point neurons allow for a novel neurobiological understanding of the integrative functions, and impairments in those functions, that were hypothesized by pioneers in the clinical care of, and research on, schizophrenia to be characteristic of the disorder. We demonstrated how multiple aspects of schizophrenia, many of which had already been usefully understood as manifestations of a widespread disturbance in context sensitivity, can be more deeply understood – both in and of themselves and as manifestations of a common processing disturbance -- by incorporating the insights of apical integration theory.

At the same time, clarification is needed on several issues. One involves a relative lack of evidence for some of the relationships we hypothesize. For example, clear relationships have not been established between levels of the neuromodulators we discussed and the perceptual, cognitive, and other psychological disturbances that we in part attribute to dysfunctional neuromodulation. There is stronger evidence for relationships between factors such as NMDA receptor hypofunction, PV and SST interneuron dysfunction, apical dendrite density reduction, and the phenomena hypothesized to result from a reduction in cooperative context sensitivity. There are also questions regarding the place of apical integration failures in schizophrenia relative to alterations in specific brain structures (e.g., striatum, claustrum, prefrontal cortex, right hemisphere) and networks that may also affect the level of coordination of cognitive activity. It is not known how these forms of coordination interact with each other, how these interactions might be dysfunctional in schizophrenia, and whether some types of dysfunctional coordination might arise more from one or another mechanism. A related issue is that this paper focused primarily on integration of apical and basal compartment activity in L5 pyramidal neurons, since much of the evidence for apical integration theory comes from studies of these neurons (Phillips, 2023). They are thus the canonical model for this theory. However, L2/3 pyramidal neurons also have relatively long apical dendrites and are thought to implement contextual modulation as well (Phillips, 2023; Larkum et al., 2007; Petousakis et al., 2023). It has also been shown that L2/3 neurons function abnormally in schizophrenia and are characterized by dendritic spine pathology, and these changes are thought to be related to cognitive deficits in the disorder (Dienel et al., 2025; Glausier and Lewis, 2013). Integration of dendritic input is less understood in L2/3, however. Moreover, there are structural and functional differences in pyramidal cells in different cortical layers (Tjia et al., 2017), and so it cannot be assumed that L5 and L2/3 pyramidal cells exhibit identical modulatory functions. The similarities and differences in L5 and L2/3 pyramidal cells, in terms of contextual modulation and its impairments in schizophrenia, is an important area for future work. Another issue in need of investigation is whether apical integration effects differ in the right versus left hemisphere, and whether any such asymmetry differs in people with schizophrenia. The possible relevance of this comes from evidence that whereas the density and size of pyramidal neurons is normally greater in the left frontal than right frontal cortex, in schizophrenia pyramidal cell density is greater in the right hemisphere and cell size is equivalent across hemispheres (Cullen et al., 2006). Whether this is related to the language, and other, disturbances discussed above is not yet known.

Furthermore, additional clarification is needed regarding the relationships between positive, negative and disorganized symptoms, along with the shared and distinct mechanisms giving rise to each. One view, echoing Bleuler's focus on loosening of associations, is that disorganization is the core syndrome of schizophrenia and that what we now call positive and negative symptoms are secondary symptoms that arise as compensations. That is, symptoms such as delusions and hallucinations represent attempts to create meaning from disorganization, while negative symptoms such as withdrawal, apathy, and catatonia reflect reductions in processing and varying levels of “shutting down” due to an inability to create meaning from disorganization. However, there is not clear evidence that for all or most people with the diagnosis of schizophrenia, disorganization is present at the outset of illness, that positive and negative symptoms are adaptations to disorganization, or that in people with chronic and deteriorating presentations, disorganization is the inevitable long-term outcome. Given the genetic variability among people with schizophrenia it appears more likely that different people with the diagnosis are affected to different degrees of severity by dysregulation in biological systems that primarily drive functions such as aberrant salience, reward sensitivity, and context-sensitivity, giving rise to positive, negative, and disorganized symptoms, respectively, which then interact with each other in ways that are still mostly not understood.

An additional issue involves the similarities and differences between the theory we propose and the predictive processing view of psychosis. The latter overlaps considerably with our view, but there are also some important areas of non-convergence. The main area of apparent difference involves predictive processing views (which are sometimes oversimplified) that emphasize that prediction error is the primary type of signal that is fed forward in neural networks, while predicted sensory signals are cancelled. In contrast, apical integration theory emphasizes the amplification of sensory information that is likely and relevant in the current context. Both views agree that information that is highly redundant (e.g., repeating stimuli across a visual field) is suppressed. A partial resolution to the seeming incompatibility between the predictive processing and apical integration theory views is that: 1) while termination of expected sensory information processing may occur, the processing of predicted stimuli nevertheless continues based on further processing of the top-down signals that matched the sensory signals; and 2) apical dendrite activity and the mechanisms discussed in Section 2 above are largely responsible for generating the top-down signals that lead to accurate representations of sensory stimuli. The validity of these hypotheses needs further study as: 1) apical integration theory has primarily focused on contextual modulation of sensory signals and not on the ways in which signals representing mismatches between contextual information and feedforward input are further processed; 2) predictive processing theory has not incorporated insights about two-point neurons and related mechanisms (e.g., dendritic coupling, the role of cortical layer 6b) into its models; and 3) both views need to better account for findings that the signaling of predicted and relevant stimuli can take the form of either stronger or weaker signals, depending on the task or situation.

There are good reasons for why schizophrenia has been referred to as ‘the graveyard of neuropathologists’ (Plum, 1972). The original meaning of this comment was that there appear to be no structural brain findings in schizophrenia that are not also found in other disorders and in healthy people. In addition, however, it is a heterogenous condition, few findings apply to the majority of people with the diagnosis (Heinrichs, 2005), and we do not understand how its structural and functional brain findings lead to the symptoms experienced by patients (Wang and Krystal, 2014). Thus, while there are many findings about schizophrenia, the search for a comprehensive understanding remains as a holy grail of psychiatry. For these reasons, it is especially important to attempt to better characterize what appear to be meaningful subtypes of patients, and/or dimensions of psychopathology, and to propose theories with testable hypotheses about observable phenomena with high reliability (Palaniyappan, 2026). The view we propose is strongly grounded in findings from neurobiology, clinical psychology, psychiatry, and computational modeling, and many findings stated or implied by earlier versions of the theory have been confirmed over the past 25 years. Most of that work has been in animal or computational studies, or studies based on healthy humans. Therefore, work is needed that tests the validity of our hypotheses on animal and computational models of schizophrenia and on people with schizophrenia. If the mechanisms we hypothesize for disorganization are supported by this evidence, an important next step will be the translation of this mechanistic understanding to the development of pharmacologic, brain stimulation, genetic, and psychological interventions to normalize apical integration and related mechanisms in people with schizophrenia. In short, even if it cannot be decided whether our understanding of schizophrenia is like a glass that is half-full or half empty, it is nevertheless important to continue to fill the glass.

## CRedIT author statement

Steven Silverstein: Conceptualization, writing, figure creation, editing, reviewing. Timothy Zolnik: Writing, figure creation, editing, reviewing. Matthew Larkum: Writing, figure creation, editing, reviewing. Judy Thompson: Writing, editing, reviewing.

## Generative AI use

Generative AI was not used in the preparation of this manuscript, but was used for creation of the graphical abstract.

## Funding

This research did not receive any specific grant from funding agencies in the public, commercial, or not-for-profit sectors.

## Declaration of competing interest

The authors declare that they have no known competing financial interests or personal relationships that could have appeared to influence the work reported in this paper.

## Data Availability

No data was used for the research described in the article.

## Footnotes

1 In addition to the presence of at least a subset of these symptoms at threshold level for at least a month (or less if treatment was initiated during that time), other diagnostic criteria include that the symptoms are not due to substance use or another known medical condition, threshold- and/or residual-level symptoms have persisted for at least 6 months, and there has been a marked decline in real-world functioning.
