# Supplementary material for: Apical integration theory provides a neural basis for understanding disorganization and consciousness in the schizophrenias
Source: Curr Res Neurobiol. 2026 Jul 24;11:100165. doi: 10.1016/j.crneur.2026.100165 (PMC13452318; doi:10.1016/j.crneur.2026.100165)
Supplement: Supplementary file 1 [file mmc1.docx]

Peer Review Overview

**Manuscript Title:** Apical Integration Theory Provides a Neural Basis for Understanding Disorganization and Consciousness in The Schizophrenias

| Received |  | Mar 26, 2026 |
| --- | --- | --- |
| 1st Decision |  | May 6, 2026 |
| 1st Revision Submitted |  | Jul 11, 2026 |
| Accepted |  | Jul 21, 2026 |

1st Decision letter

### **Reference:** CRNEUR-D-26-00006R1

### **Title:** Apical Integration Theory Provides a Neural Basis for Understanding Disorganization and Consciousness in The Schizophrenias

### **Journal:** Current Research in Neurobiology

Dear Dr. Silverstein,

Thank you for submitting your manuscript to Current Research in Neurobiology. 

I have completed my evaluation of your manuscript. The reviewers recommend reconsideration of your manuscript following major revision. I invite you to resubmit your manuscript after addressing the comments below. Please resubmit your revised manuscript by **Jun 05, 2026**. 

When revising your manuscript, please consider all issues mentioned in the reviewers' comments carefully: please outline every change made in response to their comments and provide suitable rebuttals for any comments not addressed. Please note that your revised submission may need to be re-reviewed.  

To submit a revision, go to https://www.editorialmanager.com/crneur/ and log in as an Author. You will see a menu item call Submission Needing Revision. You will find your submission record there.

When you submit the revised manuscript, please provide a separate document, uploaded as 'Detailed Response to Reviewers,' that carefully details, point-by-point, the list of changes made in response to the reviewers' comments. You may also include a suitable rebuttal to any specific request for change that you have not made. The journal strongly encourages you to submit two versions of your revised manuscript in order to facilitate the evaluation process: one with changes in the manuscript marked (upload as file type: Manuscript (REVISED, text with changes Marked)), in addition to an unmarked, production-ready version (required) (upload as file type: Manuscript).

Research Elements (optional)
This journal encourages you to share research objects - including your raw data, methods, protocols, software, hardware and more – which support your original research article in a Research Elements journal. Research Elements are open access, multidisciplinary, peer-reviewed journals which make the objects associated with your research more discoverable, trustworthy and promote replicability and reproducibility. As open access journals, there may be an Article Publishing Charge if your paper is accepted for publication. Find out more about the Research Elements journals at https://www.elsevier.com/authors/tools-and-resources/research-elements-journals?dgcid=ec_em_research_elements_email.


Current Research in Neurobiology values your contribution and I look forward to receiving your revised manuscript.

Kind regards,  

Anna S Mitchell, Ph.D. 
Editor in Chief 
Current Research in Neurobiology 

**Comments from Editors and Reviewers:**

Your paper on two-point neurons in the schizophrenias has been classified as requiring major revision, which is to be submitted within 60 days. That is the decision proposed by reviewers 1 and 3. It is seen as being of potentially major importance by reviewers 2 and 3, but Reviewer 1 is far from convinced of that. Reviewer 1 sees your paper as expressing a degree of confidence in its claims that far exceeds the strength of the grounds on which they are based. So, in this context, it is of the greatest importance to change the whole tone of the paper to one that highlights doubts such as those expressed by Reviewer 1. You should focus on presenting them as examples of how the discovery of the distinct functions of apical and basal dendrites opens the door to wide new fields of exploration, including those in the cellular neuropsychopathology of the schizophrenias. That would be in keeping with the central aim of this special issue of the journal, which is to inspire investigation of the many new issues raised by those discoveries and their interpretation as evidence for two-point neurons. Instead of, in essence, claiming that the bridge from cellular dysfunctions to psychological symptoms in the schizophrenias has now been largely built, your paper should focus on identifying rigorously specified, tractable, and unresolved issues that arise from seeing those disorders from the perspective of two-point neurons. Science is propelled by doubt, so firmly avoid the overconfidence to which you are understandably tempted by the great promise that you see in relating TPNs to the schizophrenias. That overconfidence is obviously premature until either highly specific predictions unique to a specific conception of two-point neurons has been unambiguously confirmed or transformative therapies for the shizophrenias have been engineered and shown to be highly successful. So, restrict the claims that something has been ‘demonstrated’ to few, if any, cases where there can be no justifiable doubts. The only thing that you can learn about that of which you are certain is that you were wrong. Wherever your say that some possibility is ‘likely’, which you do many times, clearly present it as an unresolved issue that now awaits investigation. That applies even more to cases where you resort to saying ‘we believe’.
All three reviewers give a high rating to the importance of the issue with which you are dealing. They have worked hard to suggest ways in which your paper could be improved. As currently presented, it is exceptionally long. The longest paper ever previously published in this journal has 217 references. In its current form yours has 378. So, do all that you can to make it shorter, and thus more user friendly. Another way to make it more friendly will be by dividing sentences that are more than three lines long into shorter sentences. That is usually possible and can sometimes reduce the number of words used. Avoid repeating things that you have already said. Be more disciplined in focussing on the key messages to be conveyed. The whole section on the striatum could be deleted, and that on dopamine reduced to a single short paragraph. Neither have a key role in your argument. Although there is value in relating your perspective to the history of speculative notions of schizophrenia, this is not the appropriate journal for doing so in any way other than by a brief, succinct, summary. In contrast to Reviewer 1, however, I recommend strengthening (and preferably without lengthening) the sections on language, conscious experience, and NMDA. I see them as a central part of the message to be conveyed. Throughout the revising process be encouraged by the high rating given by all reviewers to the topic on which you are working and focus on issues that are most relevant to readers of this journal. That will give your paper a better chance of helping to produce a widespread interest in the perspective that you advocate.
 

**Reviewer 1:**

This review by Silverstein and colleagues explores a potential link between function of "two-compartment" layer 5 pyramidal neurons (L5PNs) in the etiology of schizophrenia. The overall scope of the review is vast, but it is this vastness serves more to detract from the overall goal than enhance the quality. The writing is unfocused, wandering to multiple topics, often with little reflection on whether these aspects would interact with the functions of L5PNs or how, mechanistically, this could happen. Some sections read as if they were written piecemeal for other works and pigeonholed in this review (section 7 is particularly egregious in this regard). Writing often assumes knowledge from the reader, presenting new concepts without definition and often without context. The writing with respect to neurobiology is loose, at times flowery, and often inaccurate.

Most information presented in this paper linking complex higher order exclusively human processes to apical integration is circumstantial at best. There are far too many logical leaps to build a convincing argument that apical integration is at the heart of the disorganization theory the authors attempt to invoke. There needs to be a better description of apical integration and how the higher order processes engage this phenomenon other than (paraphrasing) "this area projects to the apical region so the apical region must be important."

The authors would benefit greatly from marked editing, placing limits on the overall length of the work. Be direct and focused. I'd estimate that 20% of this text truly relates to the concept of apical amplification and its relation to schizophrenia. That work would be far more impactful and informative if all the text was focused on the matter at hand: the potential link between cortical coincidence detection and its dysfunction in schizophrenia.

Please note that specific comments on text below are not exhaustive, but will hopefully provide a framework for improving the manuscript overall:

One major suggestion is to reformat the work to have a section that simply and concisely explains how pyramidal cells integrate information, what apical amplification is, and how these processes are supported by the structure of the neuron, the placement of specific excitatory and inhibition input streams, and the presence and distribution of various conductances along arbors. This would help contextualize discussions that occur later, focused on schizophrenia etiology, far better.
Similarly, discussions of Layer 6 neurons and thalamic loops come and go in the text. The authors define the circuit of layer 5 neurons as a hub of apical integration, but then later on almost exclusively use layer 6B neurons as their corollary for apical integration. There are far too many logical leaps to make this presentation of ideas compelling. This could be simplified again if these cell types and circuits are discussed first in a general sense, with later text elaborating upon potential roles in SCZ etiology.
The language in this text when it comes to receptors, neurons, circuits, and network activity is often anthropocentric. This approach should be eliminated when dealing with these biological topics.
References are often provided for psychology related work while there are times when basic neuroscience statements are left without support. I found myself questioning the validity of many of these claims, either due to poor phrasing or outright inaccuracies. The authors would benefit greatly from proper referencing, as well as consultation with neurophysiologists.
The figures are not very effective at advancing the author's ideas and are poorly referenced in the text. Building a flowchart or table of the different pathways and brain regions where apical integration plays a role and what that role is would be better. The graphical abstract need substantial revisions to enhance clarity and meaning. In figure 1 it is unclear what success in the task looks like and what dysfunctional responses look like. Figure 2 is similarly poorly referenced in the text and the legend is insufficient. Figure 3 would benefit from an improved legend and in C a marker for the coupling zone. Figure 4 needs to be remade. Figure 5 has fine information but again is poorly referenced in the text and the figure is not more useful than more carefully crafted text.
Similarly, the title is abstruse.
P4, the concept of "figure-ground" is raised, which may be understood well in psychology, but is less common in neuroscience. Please take the time to provide a definition.
Top of P6, there is a call to Goldstein 1934 that appears out of place.
P7, use of "nexus or fulcrum". One might want to simplify to fulcrum here, as nexus has been used by some to describe the base of the apical tuft of L5PNs.
P7: "We review evidence that cooperative context sensitivity is a function of the degree to which apical dendrite activity can modulate processing of signals input to the neuron's soma and thereby affect the firing (output) strength of the neuron."
This is written in a way that implies that synaptic input to the apical tuft is purely modulatory, but lacks contextual or other information itself. Is this what the authors intend? The next sentence captures the first-order role of the tuft more accurately; it carries the context of activity elsewhere in the brain.

P8 "The contextual modulation provided by apical dendrite activity has the function of increasing the coherence between our beliefs…" This is a proposed function; please couch these ideas appropriately.

P11, (e.g., receptive field) is not a primary signal. It is the sensory range in which the neurons can respond to stimuli.

P11, the introduction of NMDARs is quite jarring with little to no background in why it is introduced here or what the goal of its introduction is at this moment. Similarly, the introduction of the biology related to layer 5 pyramidal neurons on this page assumes quite a bit of knowledge from the reader. Strongly consider reframing this section with proper background. The concepts that the apical and basal arbors receive different input streams, that thalamocortical loops (what is a loop in this case?) are important for perception, and that dendritic nonlinearities can aid in perception, all need to be introduced.

Much of the references to NMDA hypofunction are inadequate. Regarding ketamine specifically, in one section the authors state that ketamine does not mirror schizophrenia psychosis and is instead more in line with SUD psychosis but then turn around and use ketamine mouse studies as support for their argument about NMDA hypofunction and apical integration.
The paper needs to be substantially reorganized. There is a very circuitous flow of information that makes it difficult for the reader to follow. One specific example: NMDA hypofunction is in section 9.4, but there has already been a substantial discussion of NMDA hypofunction.
P13: "It is important to emphasize that NMDA receptors are involved in many non-perceptual functions such as working memory and learning, and that they are especially dense in the prefrontal cortex (PFC) relative to posterior regions." provide citations for this statement

P15:" HCN channels are partly open when there is no synaptic input and this allows positively charged ions to enter the cell, which lowers the threshold for generating an action potential." "when there is no synaptic input" is quite odd; specify membrane voltage ranges. Also, the interaction between HCN and AP generation is complex. HCN does not have a direct effect on threshold, per se, but the presence of HCN current can affect NaV availability in the AIS, which will influence threshold. Furthermore, open HCN channels reduce membrane resistivity, which can affect the ability of an incoming synaptic input to propagate to the AIS and evoke an AP. This is not something unique to HCN channels localized to apical or basal arbors. The dichotomy presented is inaccurate.

P17, some of this text should be moved to a section describing apical amplification and L5PN function in general. This should be earlier in the overall structure of the paper.

P18, S2.6, there is a statement that could imply that L6b neurons synapse with L5b neurons via NMDAR-only synapses. This should be clarified.

S2.7, medial, not median

S2.7," orexin blocks flow through HCN channels." This is another case of very loose, and as a result, incorrect, language. Orexin has no direct effect on HCN channels, and it certainly is not a channel antagonist. Orexin receptors can modulate HCN channels via PKC-dependent signaling, which can shift HCN voltage dependence of activation.

"For example, 5-HT1 receptors cause hyperpolarization while 5-HT2 and 5-HT4, 5-HT5, 5-HT6 and 5-HT7 receptors are depolarizing [102]." Similar problem. G-protein coupled 5HT receptors are conflated with ionotropic 5-HT receptors. Fix this.

Dopamine receptor section: The authors need to do a much better job of describing the current state of the field regarding the dopamine hypothesis of schizophrenia. The authors state [paraphrased] that "It may be unknown what exactly in the mesolimbic system contributes to hallucinations", but this dismisses clinical use of D2R antagonists to alleviate positive symptoms. Also, the prevalence of hallucinations in parkinsonian patients treated with levodopa needs to be discussed.
Similarly, the authors propose a theory implicating hyperfrontality despite evidence that in SCZ there is hypofrontality.
L5b cells in PFC tend to express D2Rs exclusively, not D1Rs. How is D2R activation, and inhibition of PKA, suppressing HCN channel function?

S3.6 "Learning has its basis in synaptic plasticity, and this depends on the context sensitivity of apical dendrites. Learning also depends on attention and short-term memory, which, as noted above, also involve apical amplification." Implies that all learning involves cortex and L5PNs. This is incorrect. Further, this reviewer did not recall a section of short-term memory noted above.

S3.7 is highly speculative in relation to apical dendrites. Is it necessary? Could it at least be condensed markedly?

P37, ACh, not Ach. 5-HT not 5HT

P39, what do you mean by "DA turnover"? This is, again, loose. It could refer to dopamine itself, reuptake, receptors, diffusion…?

P40, This is one example where the authors write something as if it were an absolute truth: "an overall flattening of response probabilities caused by cortical DA reduction, novel associations and meanings can formed". First, this lacks any citations. Second, how can one ascribe a flattening of response probabilities (whatever that means) specifically to dopamine? Where is the strong evidence that such things occur?

The reader does not care if Cobenfy has a registered trademark.

Section 4 is some of the better writing in the paper, but it does not have much relevance to apical amplification concepts. It could be focused.

Similarly, while interesting and well written, the continued discussion of the language symptoms in schizophrenia in this paper are not connected to the central thesis of the work. There is insufficient evidence to link language to their apical integration theory, making the inclusion inappropriate.
Section 6: The consciousness argument is not compelling. Using these psychological terms to refer to schizophrenia as a disorder of altered consciousness is not supported by the authors' neurobiological evidence. Do people coming in and out of anesthesia present as schizophrenic? This and section 7 could be deleted.


**Reviewer 2:**

The manuscript by Silverstein et al is an interesting review which uses the two-point theory to understand disorganization and consciousness in the schizophrenias. Overall, the review demonstrates that the concepts and findings inherent to apical integration theory support the seminal understandings of schizophrenia. In particular, two-point neurons can explain the impairments of integrative functions seen in schizophrenia. The authors propose that a primary factor in the reduction of organized mental activity in schizophrenia is a reduction in the degree to which apical dendrites can modulate processing of input to the soma. This is an extensive and thorough review which may provide further insight into brain dysfunction in schizophrenia. Below are comments which may help improve the clarity of the manuscript:

This review discusses many sub-topics and often reads more like a textbook chapter than a review article. Please consider decreasing the number of different topics discussed and expand on those topics which are considered more relevant. For example, I suggest removing sub-topics which are not extensive (for example, section 3.8 only has two sentences). This will greatly enhance the clarity of the review by highlighting the most relevant information.

Please reduce the amount of text in 1.2 that describes what each section discusses as this text is redundant.

In Figure 1, the bar graphs in 1A and 1B either need to have a y axis value or included on the same axis (to allow for direct comparison). Otherwise the graphs themselves are uninformative.

The main take-home message of the review, that two-point neurons can explain the impairments of integrative functions seen in schizophrenia, should be clearly depicted in a Figure. Please either expand upon Figure 1, or have a new Figure.

At times, the text describing biology and cellular properties does not link directly to dysfunctions in schizophrenia. This makes the text disjointed and clarity would be improved if there was always this link involved in every sub-section.

This review covers two broad main topics; disorganization and consciousness (which could be separated into two different reviews). The link to disrupted consciousness in schizophrenia and two-point neurons is less clear, and this might be enhanced by a dedicated Figure.


**Reviewer 3:**

Dear editors and authors,

This piece by Silverstein and colleagues has been - genuinely - one of the most intellectually engaging I've had the pleasure to review. Provided my concerns are properly addressed, I will be very happy to recommend acceptance. P.s.: This is a very long manuscript and the deadline for revision was a bit short, so the authors may feel encouraged to underscore any potential inconsistencies in my overall impressions of the manuscript.

Major (essential)
1. Manuscript length and Section 2
My opinion is that section 2 should be radically reduced and simplified. There are already a number of available papers on these topics that the reader may be referred to for better context or further detail. Given the already excessive amount of content we're dealing with in this manuscript, I'd strongly suggest the focus of section 2 to be conveying only what is strictly essential for readers to understand the core ideas of the manuscript. Having read all of it, I fail to see the need for such detailed explanations on topics that - in light of the focus of the actual manuscript (viz. schizophrenia) - may actually deter readers from even starting to read it. This seems especially important given the fact that it's such an introductory section, as it seems to break the natural flow of the manuscript and may substantially undermine its overall impact.
All in all, the idea would be to narrow this section down to the core principles that need to be conveyed, and how they relate to your hypotheses. It'd likely boost clarity and engagement.

2. Biomarkers and robust biological alterations
Any theoretical framework that proposes itself to provide a strong take on the underlying biological mechanisms of schizophrenia/thought disorder should probably be able to accommodate some of its chief and more well-established biomarkers. I found the discussions on some of the foremost ones (e.g., mismatch negativity, prepulse inhibition, gamma-band alterations, parvalbumin interneuron and perineural net alterations, early-ontogeny hippocampal hyperactivity) at times lacking in substance/accuracy (e.g., gamma oscillations are chiefly regulated by PV+ interneurons which carry out perisomatic inhibition, yet the authors at one point seem to speculate these to arise mainly from the apical dendrite) or (potentially) missing altogether. While it's up to the authors which and how many specific biomarkers they'd like to discuss, I strongly recommend discussing some of them in further depth.
Finally and in the same vein, at various times the authors make speculations on the nature of the pathophysiological alterations in schizophrenia which could be potentially backed - even if indirectly - by hard data on schizophrenia-specific alterations. These may involve cortical microstructure, neurophysiology and whatnot. At times this piece reads overly speculative (which seems natural given the sheer volume of - highly interesting, I should stress - ideas), so it'd be very important to make it sound more falsifiable and empirically-grounded given the extant schizophrenia literature. Suggested literature:
2.1. E/I imbalance and interneurons
Critically, a major concern was the lack of more detailed discussions on E/I imbalance in schizophrenia, especially in what pertains to interneurons. For instance, Parvalbumin+ and Somatostatin+ interneurons, which heavily influence basal and apical compartments of pyramidal cells, have repeatedly been associated with altered function in Sz. These cells play a critical (protagonistic even) role in synchronising the cortical network to gamma and theta oscillatory rhythms (respectively), for example - as well as many of the ionic dynamics underlying the pathophysiological mechanisms you propose. Hence, it seems quite opportune to further explore the modulatory roles of PV+ and SST+ interneurons on apical and basal function. Suggested literature: Almeida and Radanovic, 2022;
2.2. Role of layers II/III
Layers II/III are one of the most obvious alterations in schizophrenia's cortical structure, yet I'm not sure I recall seeing any mention of this in the manuscript. It is my opinion that this should be either included as a fulcral topic of your model, or alternatively (but less desirably, in my view) adequately addressed as a caveat. I find it unlikely that so many different features of the disease can be explained in real depth if layers II/III are overlooked. Hence, I'd ask the authors: how do you believe layers ii/iii relate to the framework you're proposing?

Minor (irrelevant for acceptance)
1. Congenital blindness: At one point the authors seem to express being somewhat puzzled by the severity of auditory alterations in sz
"Impairments in both visual and auditory
selective attention have been demonstrated in schizophrenia, although, interestingly, these appear
to be more severe in the auditory domain [129,130].".
I'd suggest looking into literature about congenital blindness and schizophrenia. If nothing else, it may be very informative: there is a striking scarcity of reported cases of congenitally blind individuals who have developed SZ. This is quite likely to involve compensatory plasticity of the auditory cortex during early development, which in turn should enhance auditory discrimination (e.g., MMN amplitudes) as well as aspects of language function which are directly related to thought disorder, inter alia. What thoughts do the authors have on this?
2. 5HT2A receptors are at one point mentioned. Cortically, these are densely expressed on the apical dendrites of layer Vb pyramids - yet, they noticeably have a depolarising effect. It would be useful for the authors to better clarify this relationship and how it might or not endorse their model.
3. The authors have underscored disintegration of perceptual and other representations in schizophrenia. In vision, SST+ interneurons seem to provide widespread divisive inhibition (eg surround suppression) which can synchronise large ensembles of neurons in a way that may be congruent with the establishment of this "unity" - basically by modulating the activity of apical dendrites of multiple pyramids, radially. Might SST+ hypofunction be related to this effect in schizophrenia?
4. The authors mention reduced drive to the apical dendrite. However, there is also reduced inhibition. Might this hint at a reduced overall dynamic range of apical amplification/suppression in schizophrenia, and hence a more inflexible (and thus context-insensitive) state of weak disinhibition of apical dendrites?
5. Intrusive memories: Almeida (2024) proposes that the disinhibition of apical dendrites from SST+ influence is most likely the core reason driving mnemonic interference/intrusions, which seems to directly relate to some of your claims on intrusive memories in SZ. Any thoughts on this?

Key takeaway to the authors and editor

My assessment is that this manuscript is in fact highly valuable, though I believe it might need polishing - especially in terms of further discussion and integration of real-world, schizophrenia-specific data (e.g., cortical microstructure, neuroanatomy, electrophysiological biomarkers, current models etc). These more robust findings should ideally be discussed and integrated into any theoretically bold and useful model of schizophrenia - after all, the model has to accomodate the data. Altogether and to put it more plainly: at times it felt like the content gravitates more into discussing DIT than the pathophysiology of schizophrenia/thought disorder itself (e.g., as per my comments on the lack of discussion of critical aspects of the disaese, such as like mismatch negativity, layers II/III, neuroanatomical data, PV+ disinhibition and perineural nets etc, whereas anything DIT-related appeared to be discussed in far greater detail).


References (potentially useful, fully optional)
Palaniyappan, L., Homan, P., & Alonso-Sanchez, M. F. (2023). Language network dysfunction and formal thought disorder in schizophrenia. Schizophrenia Bulletin, 49(2), 486-497.
Almeida, V. N. (2024). Somatostatin and the pathophysiology of Alzheimer's disease. Ageing Research Reviews, 96, 102270.
Almeida, V. N., & Radanovic, M. (2021). Semantic priming and neurobiology in schizophrenia: A theoretical review. Neuropsychologia, 163, 108058.
Morgan, V. A., Clark, M., Crewe, J., Valuri, G., Mackey, D. A., Badcock, J. C., & Jablensky, A. (2018). Congenital blindness is protective for schizophrenia and other psychotic illness. A whole-population study. Schizophrenia research, 202, 414-416.
Javitt, D. C., Lee, M., Kantrowitz, J. T., & Martinez, A. (2018). Mismatch negativity as a biomarker of theta band oscillatory dysfunction in schizophrenia. Schizophrenia research, 191, 51-60.
Lakatos, P., O'Connell, M. N., Barczak, A., McGinnis, T., Neymotin, S., Schroeder, C. E., ... & Javitt, D. C. (2020). The thalamocortical circuit of auditory mismatch negativity. Biological psychiatry, 87(8), 770-780.
Javitt, D. C., Steinschneider, M., Schroeder, C. E., & Arezzo, J. C. (1996). Role of cortical N-methyl-D-aspartate receptors in auditory sensory memory and mismatch negativity generation: implications for schizophrenia. Proceedings of the National Academy of Sciences, 93(21), 11962-11967.
Steullet, P., Cabungcal, J. H., Coyle, J., Didriksen, M., Gill, K., Grace, A. A., ... & Do, K. (2017). Oxidative stress-driven parvalbumin interneuron impairment as a common mechanism in models of schizophrenia. Molecular psychiatry, 22(7), 936-943.
Santos-Silva, T., dos Santos Fabris, D., de Oliveira, C. L., Guimarães, F. S., & Gomes, F. V. (2024). Prefrontal and hippocampal parvalbumin interneurons in animal models for schizophrenia: a systematic review and meta-analysis. Schizophrenia Bulletin, 50(1), 210-223.

1st Author Response Letter

**Response to comments from Editors and Reviewers:**

We thank the review editor and the three reviewers for their thoughtful reading of our manuscript, and for their insightful comments regarding how to improve the paper. Below, we provide a point by point response to each comment. The original comments have been italicized and our responses are in non-italicized text preceded by ‘***.’

**Editor:** *Your paper on two-point neurons in the schizophrenias has been classified as requiring major revision, which is to be submitted within 60 days. That is the decision proposed by reviewers 1 and 3. It is seen as being of potentially major importance by reviewers 2 and 3, but Reviewer 1 is far from convinced of that. Reviewer 1 sees your paper as expressing a degree of confidence in its claims that far exceeds the strength of the grounds on which they are based. So, in this context, it is of the greatest importance to change the whole tone of the paper to one that highlights doubts such as those expressed by Reviewer 1. You should focus on presenting them as examples of how the discovery of the distinct functions of apical and basal dendrites opens the door to wide new fields of exploration, including those in the cellular neuropsychopathology of the schizophrenias. That would be in keeping with the central aim of this special issue of the journal, which is to inspire investigation of the many new issues raised by those discoveries and their interpretation as evidence for two-point neurons. Instead of, in essence, claiming that the bridge from cellular dysfunctions to psychological symptoms in the schizophrenias has now been largely built, your paper should focus on identifying rigorously specified, tractable, and unresolved issues that arise from seeing those disorders from the perspective of two-point neurons. Science is propelled by doubt, so firmly avoid the overconfidence to which you are understandably tempted by the great promise that you see in relating TPNs to the schizophrenias. That overconfidence is obviously premature until either highly specific predictions unique to a specific conception of two-point neurons has been unambiguously confirmed or transformative therapies for the schizophrenias have been engineered and shown to be highly successful. So, restrict the claims that something has been ‘demonstrated’ to few, if any, cases where there can be no justifiable doubts. The only thing that you can learn about that of which you are certain is that you were wrong. Wherever your say that some possibility is ‘likely’, which you do many times, clearly present it as an unresolved issue that now awaits investigation. That applies even more to cases where you resort to saying ‘we believe’.*

***We have made many edits to the manuscript to emphasize that the points we make in the paper are to suggest hypotheses regarding the neurobiological basis of disorganization in schizophrenia, and to note that the biology-symptom links we discuss are potential realities and not ones that can be considered to be convincingly demonstrated. This includes removing all instances of “we believe” and removal of all instances of the word “likely” with respect to our hypotheses. We do not wish to claim, or be perceived as claiming, that these issues are settled. Rather, our goal is to open the eyes of the reader to the potential usefulness of applying insights from apical integration theory to the understudied area of disorganization in schizophrenia.

*All three reviewers give a high rating to the importance of the issue with which you are dealing. They have worked hard to suggest ways in which your paper could be improved. As currently presented, it is exceptionally long. The longest paper ever previously published in this journal has 217 references. In its current form yours has 378. So, do all that you can to make it shorter, and thus more user friendly. Another way to make it more friendly will be by dividing sentences that are more than three lines long into shorter sentences. That is usually possible and can sometimes reduce the number of words used. Avoid repeating things that you have already said. Be more disciplined in focussing on the key messages to be conveyed.*

***We have shortened the manuscript by 30%. The resubmitted version is 20 pages shorter than the original submission, even after adding text in response to reviewer comments. The changes to the text have also reduced the number of citations from 378 to 315. We removed over 100 citations from the original manuscript, but the final reduction was 63 because we were asked by the reviewers to provide citations for a number of points where no citation was given in the original submission, or specific references were suggested by reviewers for us to add, or we were asked to add material on specific points for which citations were required. We realize that this paper contains many references, but we view this as due to needing to cite many papers related to apical integration theory, as well as many papers on a wide range of perceptual, cognitive, and phenomenological aspects of schizophrenia.

*The whole section on the striatum could be deleted, and that on dopamine reduced to a single short paragraph. Neither have a key role in your argument.*

***We have removed the section on the striatum, and in fact have removed the entire section on other biological structures and issues that could affect cooperative context sensitivity. We now note very briefly in the discussion, in 1-2 sentences, that an issue for future research is to clarify the ways in which coordination of brain and mental activity is implemented by these brain structures or networks.

*Although there is value in relating your perspective to the history of speculative notions of schizophrenia, this is not the appropriate journal for doing so in any way other than by a brief, succinct, summary. In contrast to Reviewer 1, however, I recommend strengthening (and preferably without lengthening) the sections on language, conscious experience, and NMDA. I see them as a central part of the message to be conveyed.*

***This section has been shortened. The summary of older views of schizophrenia that anticipated the hypothesis of reduced cooperative context sensitivity places the current neurobiological research within a historical context. This context also demonstrates the importance of the current neurobiological research in the sense of showing that it can address, in many ways for the first time, the root causes of some of the most disabling, but least understood aspects of the disorder. We wish to emphasize here that these ideas are more than just speculative notions of schizophrenia. They are some of the founding ideas about what schizophrenia is at the most fundamental level (an issue on which consensus has still not been reached, even in 2026, hence their importance), they are supported by decades of clinical, neuropsychological, and genetic evidence, and the authors we have cited are frequently cited in the psychiatric literature even today. Nevertheless, we have removed non-essential text from this section (text that did not directly relate to disorganization) and the entire discussion of historical precursors is now just slightly over 1 page of double-spaced text. Regarding the sections on language, consciousness and NMDA receptor activity, we believe these are all now stronger. Much of this is due to the feedback we received from the reviewers, including adding additional points and citations.

*Throughout the revising process be encouraged by the high rating given by all reviewers to the topic on which you are working and focus on issues that are most relevant to readers of this journal. That will give your paper a better chance of helping to produce a widespread interest in the perspective that you advocate.*

***We agree that the topic is important, and the revised manuscript is more focused on a smaller subset of issues on that topic. We have removed a number of sections of the paper, including those that were least relevant to readers of this journal (e.g., the section on psychotherapy, the redundant text on the structure and focus of the paper in the Introduction, the section on other possible biological factors involved in mental organization, and the section on surround suppression). We have also eliminated redundant text in many cases.

***Reviewer 1:*** *This review by Silverstein and colleagues explores a potential link between function of "two-compartment" layer 5 pyramidal neurons (L5PNs) in the etiology of schizophrenia. The overall scope of the review is vast, but it is this vastness serves more to detract from the overall goal than enhance the quality. The writing is unfocused, wandering to multiple topics, often with little reflection on whether these aspects would interact with the functions of L5PNs or how, mechanistically, this could happen. Some sections read as if they were written piecemeal for other works and pigeonholed in this review (section 7 is particularly egregious in this regard). Writing often assumes knowledge from the reader, presenting new concepts without definition and often without context. The writing with respect to neurobiology is loose, at times flowery, and often inaccurate.*

***We have shortened the paper to improve its focus, and have significantly edited all remaining sections to improve clarity and accuracy. Sections 7 (on psychotherapy) and 8 (on other brain structures and networks that might be involved in disorganization in schizophrenia) have been completely removed, with the latter issue now only referred to briefly in the Discussion. Similarly, some of the questions/subsections in the Questions Arising section were seen (upon further reflection) as straying too far from the main arguments of the paper and these have been removed. The entire Questions Arising section has actually been removed but the three questions/subsections that we believe were most important have been moved to other parts of the paper. The paper now has only 4 major sections, along with a relatively brief Discussion. These sections are: 1) Introduction: The schizophrenias and the search for unifying neurobiological mechanisms; 2) A neural basis of disorganization in the schizophrenias; 3) Schizophrenia-related impairments that suggest alterations in apical dendrite function and associated mechanisms; and 4) The nature of disturbed consciousness in the schizophrenias.

*Most information presented in this paper linking complex higher order exclusively human processes to apical integration is circumstantial at best. There are far too many logical leaps to build a convincing argument that apical integration is at the heart of the disorganization theory the authors attempt to invoke. There needs to be a better description of apical integration and how the higher order processes engage this phenomenon other than (paraphrasing) "this area projects to the apical region so the apical region must be important."*

***Apical integration is a relatively new discovery, and we acknowledge that there is limited data in humans for its involvement in the perceptual, cognitive, and behavioral processes we discuss. Furthermore, there are no data showing direct links with the aspects of schizophrenia that are our focus. In revising the manuscript, we have made sure to note these points. In addition, we have extensively edited the paper so that what are promising avenues to explore are clearly delineated as such, and we have removed all of the instances where we previously referring to links with schizophrenia as “likely.” At the same time, despite the fact that much of the evidence for apical integration theory comes from animal and computational modeling studies, the evidence from these studies is often used to theorize about perception, cognition, and behavior in humans in existing literature. We view our discussion of schizophrenia as both an extension of this work, and as an extension of our prior work on impaired cognitive coordination and NMDA receptor hypofunction in schizophrenia. That is, we believe apical integration theory provides a solid biological foundation for these now-accepted phenomena in schizophrenia. Because the biological basis of schizophrenia remains poorly understood, and because its heterogeneity remains understudied (although it is commonly acknowledged to exist) we believe it is important to highlight the ways in which apical integration theory *might* provide a useful framework for understanding the most disabling symptom cluster in the disorder (i.e., disorganization). In the spirit of Reviewer 1’s comments then, we now more clearly acknowledge that our goal is to point the way towards future research rather than to confirm a theory. In addition, we have removed or substantially edited specific comments that might be perceived as overly speculative, or too far removed from the apical integration process, including several whole sections. We have also removed sentences that resemble those the reviewer described at the end of the comment above.

*The authors would benefit greatly from marked editing, placing limits on the overall length of the work. Be direct and focused. I'd estimate that 20% of this text truly relates to the concept of apical amplification and its relation to schizophrenia. That work would be far more impactful and informative if all the text was focused on the matter at hand: the potential link between cortical coincidence detection and its dysfunction in schizophrenia.*

***Based on the comments of Reviewer 1 and the other two reviewers, the manuscript has been shortened by 20 pages (30%). We believe this has significantly improved the paper. We disagree with Reviewer 1, however, that the paper would be more impactful if it was focused only on cortical coincidence detection and its dysfunction in schizophrenia. While such a paper would be useful, our goal is to demonstrate that multiple aspects of schizophrenia can be viewed as manifestations of a widespread impairment in apical integration. These phenomena are not often viewed as being related to each other, and for many of them, no compelling biological basis has been proposed in the literature. In other cases, functions hypothesized to be the basis of much of the perceptual and cognitive impairments in schizophrenia (e.g., predictive coding failures) do not appear to account for them as well as does impaired apical integration. To convey these points, a focus broader than coincidence detection is necessary. Our goal is to provide schizophrenia researchers and clinicians with a new perspective on many of the most difficult-to-treat aspects of the disorder (e.g., symptoms related to disorganization in its many forms).

*Please note that specific comments on text below are not exhaustive, but will hopefully provide a framework for improving the manuscript overall:
One major suggestion is to reformat the work to have a section that simply and concisely explains how pyramidal cells integrate information, what apical amplification is, and how these processes are supported by the structure of the neuron, the placement of specific excitatory and inhibition input streams, and the presence and distribution of various conductances along arbors. This would help contextualize discussions that occur later, focused on schizophrenia etiology, far better.*

***The revised paper has been extensively edited and reorganized. We have also revised all of the figures and added 3 new ones, to help clarify points about neurobiology (e.g., the function of L5 pyramidal neurons; L6b and cortico-thalamic loops) and the relationships between the varied phenomena in schizophrenia that we believe have their basis in apical integration impairments. The remaining four major sections are listed in a comment above. We hope the revised text is seen as a more effective presentation than our prior version of it.

*Similarly, discussions of Layer 6 neurons and thalamic loops come and go in the text. The authors define the circuit of layer 5 neurons as a hub of apical integration, but then later on almost exclusively use layer 6B neurons as their corollary for apical integration. There are far too many logical leaps to make this presentation of ideas compelling. This could be simplified again if these cell types and circuits are discussed first in a general sense, with later text elaborating upon potential roles in SCZ etiology.*

***We emphasize the importance of both layer 5 and layer 6 neurons, but also highlight the distinct roles they play. For example, by receiving both sensory input (at the level of the cell soma) and contextual input (in their dendrites in layer 1), layer 5 neurons are critical for implementing contextual modulation of sensory and cognitive activity. Our emphasis on layer 6 neurons is on their neuromodulatory effects on arousal, and on the effects of changes in arousal on the extent of context sensitivity of layer 5 neurons. We have now clarified this emphasis in the paper.

*The language in this text when it comes to receptors, neurons, circuits, and network activity is often anthropocentric. This approach should be eliminated when dealing with these biological topics.*

***We identified several instances of this in the paper and have revised or removed these sentences.

*References are often provided for psychology related work while there are times when basic neuroscience statements are left without support. I found myself questioning the validity of many of these claims, either due to poor phrasing or outright inaccuracies. The authors would benefit greatly from proper referencing, as well as consultation with neurophysiologists.*

***In the context of an overall effort to reduce the number of references, we have added additional references for biological findings that did not have accompanying citations in the earlier version of the manuscript. We also note that two of the four authors (ML and TZ) are neurophysiologists and we have, as a group, carefully reviewed all of the statements in the manuscript and removed or modified any that could be perceived as inaccurate or ambiguous.

*The figures are not very effective at advancing the author's ideas and are poorly referenced in the text. Building a flowchart or table of the different pathways and brain regions where apical integration plays a role and what that role is would be better. The graphical abstract need substantial revisions to enhance clarity and meaning. In figure 1 it is unclear what success in the task looks like and what dysfunctional responses look like. Figure 2 is similarly poorly referenced in the text and the legend is insufficient. Figure 3 would benefit from an improved legend and in C a marker for the coupling zone. Figure 4 needs to be remade. Figure 5 has fine information but again is poorly referenced in the text and the figure is not more useful than more carefully crafted text.
Similarly, the title is abstruse.*

***Based on your comment, we have changed to title to: “Apical Integration Theory Provides a Neural Basis for Understanding Disorganization and Consciousness in The Schizophrenias”. We believe this sends a clearer message regarding what the paper is about. Regarding the figures, we have made the following changes:

- Simplified and revised Figure 1 and its description
- Added a new Figure 2, to visually depict the different locations of key L5 features and their functions, such as the apical dendrites that are the sources of contextual input, the oblique dendrite region that functions as a coupling zone that gates the influence of tuft activity on neuronal output and the basal dendrites that receive sensory (or other feedforward) input
- Figure 3 is the former Figure 2 (and is now easier to interpret due to the addition of the new Figure 2). Requested modifications to this figure have been made
- Figure 4 is the former Figure 3. Minor changes have been made to this figure and the figure caption has been improved
- Figure 5 is entirely new. This figure shows the relationship between Layer 6b functions and cortico-thalamic loops
- Figure 6 is a revision of the former Figure 4. In addition to showing the different manifestations of disorganization in schizophrenia, it now also depicts the biological mechanisms whose impairments are thought to lead to disorganization
- Figure 7 is a complete revision of the former Figure 5, on the contour integration task and data from people with schizophrenia. The new figure includes additional stimulus examples from the task, and a new figure with psychometric functions from patients and control subjects. The caption explains the findings and notes their support in ERP and fMRI studies using the task with schizophrenia patients. The revised figure more clearly makes the point about reduced integration in perception in schizophrenia compared to the former Figure 5
- Figure 8 is new and shows the different manifestations of disturbed consciousness in schizophrenia (this was also added in response to a comment by Reviewer 2)
- The graphical abstract was completely redone

***Thank you for the suggestions about revising the figures.

*P4, the concept of "figure-ground" is raised, which may be understood well in psychology, but is less common in neuroscience. Please take the time to provide a definition.*

***Thank you for pointing this out. We now provide a definition, and have also split the long sentence in which the term was embedded into multiple sentences to improve flow and readability.

*Top of P6, there is a call to Goldstein 1934 that appears out of place.*

***The idea that symptoms in brain disorders (including schizophrenia) represent substitute and simplified models of reality, allowing the patient to cope with less anxiety, albeit it in less effective ways in some respects, was correctly attributed to Goldstein. However, the punctuation used in the original sentence rendered the Goldstein citation ambiguous. This sentence, and the following one, were initially edited to clarify this point. However, as part of the effort to reduce the amount of historical context in the paper, especially that which is not directly related to disorganization, the section of text that included this comment has been deleted from the paper.

*P7, use of "nexus or fulcrum". One might want to simplify to fulcrum here, as nexus has been used by some to describe the base of the apical tuft of L5PNs.*

***We have removed this text as part of removing several pages in the Introduction to improve the flow of the opening statements.

*P7: "We review evidence that cooperative context sensitivity is a function of the degree to which apical dendrite activity can modulate processing of signals input to the neuron's soma and thereby affect the firing (output) strength of the neuron."
This is written in a way that implies that synaptic input to the apical tuft is purely modulatory, but lacks contextual or other information itself. Is this what the authors intend? The next sentence captures the first-order role of the tuft more accurately; it carries the context of activity elsewhere in the brain.*

***Thank you for catching this. The sentence you mention is indeed redundant. It has been deleted, and the following sentence has been edited to note that it is the contextual information provided by layer 1 apical dendrites that allows pyramidal cells to adapt the salience of their output based on the context of activity elsewhere in the brain.

*P8 "The contextual modulation provided by apical dendrite activity has the function of increasing the coherence between our beliefs…" This is a proposed function; please couch these ideas appropriately.*

***We now note that this is a proposed function.

*P11, (e.g., receptive field) is not a primary signal. It is the sensory range in which the neurons can respond to stimuli.*

***The sentence now reads: “In sensory areas, these operations separate a neuron's primary signal (e.g., that coming from its receptive field) from contextual input that modulates the signal salience without altering its content.”

*P11, the introduction of NMDARs is quite jarring with little to no background in why it is introduced here or what the goal of its introduction is at this moment. Similarly, the introduction of the biology related to layer 5 pyramidal neurons on this page assumes quite a bit of knowledge from the reader. Strongly consider reframing this section with proper background. The concepts that the apical and basal arbors receive different input streams, that thalamocortical loops (what is a loop in this case?) are important for perception, and that dendritic nonlinearities can aid in perception, all need to be introduced.*

***The introduction of NMDA receptors occurs after the discussion of the concept of contextual modulation to indicate both that NMDA receptor function is thought to be involved in implementing contextual modulation and that NMDA receptor hypofunction in schizophrenia has been hypothesized to be the basis of reduced contextual modulation in that disorder. We then indicate that, in the case of schizophrenia, there are problems with this view, but that the discoveries related to apical integration theory point to a set of mechanisms that interact with NMDA receptor functions and that can potentially account for a number of the manifestations of disorganization that were previously hypothesized (by us and others) to involve NMDA receptor hypofunction alone. Also, in this revision, we now provide additional explanation about the proposed role of NMDA receptors in context processing, and about why earlier findings of NMDA receptor hypofunction in schizophrenia could be interpreted as being a basis for reduced context processing and the presence of disorganized (i.e., out of context) cognition and behavior. We have also removed much of the text before and after introducing these points so that these ideas are more closely linked to our primary points.

*Much of the references to NMDA hypofunction are inadequate. Regarding ketamine specifically, in one section the authors state that ketamine does not mirror schizophrenia psychosis and is instead more in line with SUD psychosis but then turn around and use ketamine mouse studies as support for their argument about NMDA hypofunction and apical integration.*

***This is a mischaracterization of the text, which states: “The idea that the acute psychotic state differs in important ways from chronic psychosis is consistent with data from ketamine (an NMDA receptor antagonist) studies. For example, one study showed a hyperconnectivity within the PFC that correlates with the extent of psychotic symptom emergence after drug use, and that was similar to findings in people at high risk for psychosis and people with a recent first psychotic episode, but not in patients with chronic schizophrenia.” This text follows a brief discussion noting that in the early stages of psychosis, when symptoms such as delusions are being formed, there appears to be an increase in novel and non-normative association formation, and that the associations that are formed may persist even into the chronic phase of psychotic illness, when hyper-excitability is no longer present. We also note that in the chronic phase of illness, these unusual associations may persist due to functional disconnection between cortical regions, including NMDA receptor hypofunction, which reduces the ability of new contextual information to modulate strongly held beliefs. The study by Anticevic et al. is considered strong evidence for this view, but we have now added description of two additional studies that also support the hypothesis. In short, in this section we discuss the idea that there is distinction between what happens during the initial stage of psychotic symptom formation and the later stages of schizophrenia and note the most relevant biological evidence for this distinction, as a way to account for the clinical evidence presented immediately prior to this text. We also wish to note here that we introduce the paragraph in question with a statement about this distinction: “While the above situation is thought to characterize many people with chronic schizophrenia, people in the earliest stages of psychotic illness (i.e., with an emerging and as yet untreated first episode of psychosis) may be characterized in some cases by excessive sensory activity [due, e.g., to active neuroinflammation, thickening of retinal neural layers, phase-related increases in glutamate levels, etc.], which may account for findings such as increased contrast sensitivity and hypersensitivity in face perception in the early phases of illness. There may also be later periods of excessive sensory activity during relapses that can be superimposed upon a progressive neurodegenerative process, consistent with the hypothesis of Phillips noted above.”

*The paper needs to be substantially reorganized. There is a very circuitous flow of information that makes it difficult for the reader to follow. One specific example: NMDA hypofunction is in section 9.4, but there has already been a substantial discussion of NMDA hypofunction.*

***We have now done much to reorganize the paper. This includes removing several sections, cutting text in many places, and moving text. As part of this, the discussion of NMDA receptor hypofunction that was in section 9.4 has been cut.

*P13: "It is important to emphasize that NMDA receptors are involved in many non-perceptual functions such as working memory and learning, and that they are especially dense in the prefrontal cortex (PFC) relative to posterior regions." provide citations for this statement*

***Citations have been added for these points.

*P15:" HCN channels are partly open when there is no synaptic input and this allows positively charged ions to enter the cell, which lowers the threshold for generating an action potential." "when there is no synaptic input" is quite odd; specify membrane voltage ranges. Also, the interaction between HCN and AP generation is complex. HCN does not have a direct effect on threshold, per se, but the presence of HCN current can affect NaV availability in the AIS, which will influence threshold. Furthermore, open HCN channels reduce membrane resistivity, which can affect the ability of an incoming synaptic input to propagate to the AIS and evoke an AP. This is not something unique to HCN channels localized to apical or basal arbors. The dichotomy presented is inaccurate.*

***We have edited and rewritten this section for accuracy.

*P17, some of this text should be moved to a section describing apical amplification and L5PN function in general. This should be earlier in the overall structure of the paper.*

***We agree and we have moved the first 4.5 lines of this section to an earlier section (formerly on page 12), where the concept of layer 5 pyramidal cells with two zones of input is first mentioned.

*P18, S2.6, there is a statement that could imply that L6b neurons synapse with L5b neurons via NMDAR-only synapses. This should be clarified.*

***This statement has been rewritten for accuracy and clarity.

*S2.7, medial, not median*

***Thank you for catching this. It has been corrected.

*S2.7," orexin blocks flow through HCN channels." This is another case of very loose, and as a result, incorrect, language. Orexin has no direct effect on HCN channels, and it certainly is not a channel antagonist. Orexin receptors can modulate HCN channels via PKC-dependent signaling, which can shift HCN voltage dependence of activation.*

***We now clarify that the effects of orexin on HCN channels are the result of activation of phospholipase C and protein kinase C (PKC) pathways, and cite a paper to this effect.

*"For example, 5-HT1 receptors cause hyperpolarization while 5-HT2 and 5-HT4,*

*5-HT5, 5-HT6 and 5-HT7 receptors are depolarizing [102]." Similar problem. G-protein coupled 5HT receptors are conflated with ionotropic 5-HT receptors. Fix this.*

***For the sake of readability, and because we wanted to shorten the section on neuromodulators, and did not want to introduce further detail that is not directly related to the apical integration hypothesis, we have deleted this sentence.

*Dopamine receptor section: The authors need to do a much better job of describing the current state of the field regarding the dopamine hypothesis of schizophrenia. The authors state [paraphrased] that "It may be unknown what exactly in the mesolimbic system contributes to hallucinations", but this dismisses clinical use of D2R antagonists to alleviate positive symptoms. Also, the prevalence of hallucinations in parkinsonian patients treated with levodopa needs to be discussed.*

***The text to which Reviewer 1 refers has been deleted since it was part of the section on other biological factors that could affect coordination of brain and mental activity. Here, we note only that while a relationship between mesolimbic dopamine and positive symptoms has been established (in schizophrenia and in Parkinson’s disease), what we were referring to was that it is still not clear how excessive dopamine leads to the subjective experience of hallucinations.

*Similarly, the authors propose a theory implicating hyperfrontality despite evidence that in SCZ there is hypofrontality. L5b cells in PFC tend to express D2Rs exclusively, not D1Rs. How is D2R activation, and inhibition of PKA, suppressing HCN channel function?*

***We have added description of additional findings to clarify this point. We now clarify that hyperconnectivity within the frontal lobe, within the default mode network, and within a cerebellar-thalamic-cortex network have been linked to disorganization in people with an emerging psychotic disorder and in people with schizophrenia. We cite three studies as evidence for this, and for the hypothesis that this disturbance is related to the initial formation of psychotic symptoms. We also cite evidence that in chronic schizophrenia there is reduced connectivity (consistent with the overall hypothesis of the paper) and that this is a mechanism whereby the abnormal neural connections formed during the initial phases of psychotic illness can remain relatively uninfluenced by activity elsewhere in the brain. Note that this evidence is consistent with other evidence that brain lesions and other insults that reduce network activity can lead to compensatory hyperconnectivity. Regarding DA receptor activity, we have explicitly removed the text related to the mechanistic issue referred to by Reviewer 1. We note that dopamine effects on L5-ET apical integration are receptor-, cell-type- and state-dependent. We have kept in points about dopamine dysregulation in schizophrenia, but do not hinge it on a specific D2R-HCN mechanism.

*S3.6 "Learning has its basis in synaptic plasticity, and this depends on the context sensitivity of apical dendrites. Learning also depends on attention and memory, which, as noted above, also involve apical amplification." Implies that all learning involves cortex and L5PNs. This is incorrect. Further, this reviewer did not recall a section of short-term memory noted above.*

***We have revised this text to indicate that synaptic plasticity is one of the neurobiological processes through which learning takes place, and that this involves context sensitivity. We also have deleted “short-term” to indicate that multiple forms of memory are involved in learning. In our discussion of memory in schizophrenia in an earlier section, we focus on those that are thought to involve reduced context sensitivity, but we also note aspects of short-term memory that are thought to involve context processing (e.g., effects of conceptual relationships in recall or word lists) that are impaired in schizophrenia.

*S3.7 is highly speculative in relation to apical dendrites. Is it necessary? Could it at least be condensed markedly?*

***Is Reviewer 1 referring to the former Section 3.8, on language and thought in schizophrenia? On the advice of the review editor, we have kept this section in the paper and have edited it, including adding and deleting text, to increase clarity. We also acknowledge that this section is more speculative than some of the other sections. At the same time, language and thought disturbances in schizophrenia are at the core of the disorganization syndrome, and one goal of the paper is to point towards a link between disorganization in perception and cognition (e.g., memory) on the one hand, and disorganization in language and thought on the other. We specifically note in one of the early paragraphs in this section that: “Phillips noted that the dependence of semantic memory and language organization on apical dendrites has not been demonstrated to the extent that it has for other domains such as perceptual binding, selective attention and disambiguation. Data on language processing in schizophrenia can inform this issue, as it demonstrates that language organization in schizophrenia is characterized by reduced contextual modulation and increased randomness, as is the case in perception and memory, where support for involvement of apical integration is stronger.” As we note above, we are clearer in the revised manuscript that our emphasis is on aspects of schizophrenia that may be fruitfully accounted for by the findings on apical integration, and that we are not implying that these relationships have been strongly demonstrated yet.

*P37, ACh, not Ach. 5-HT not 5HT*

***These errors have been corrected.

*P39, what do you mean by "DA turnover"? This is, again, loose. It could refer to dopamine itself, reuptake, receptors, diffusion…?*

***The word “turnover” has been replaced by “availability.” Complete precision is not possible here because there is not general agreement regarding how much the excess DA in schizophrenia is due to each of the factors mentioned by Reviewer 1.

*P40, This is one example where the authors write something as if it were an absolute truth: "an overall flattening of response probabilities caused by cortical DA reduction, novel associations and meanings can formed". First, this lacks any citations. Second, how can one ascribe a flattening of response probabilities (whatever that means) specifically to dopamine? Where is the strong evidence that such things occur?*

***The term “flattening of response probabilities” has been removed from the paper. It has been used in several theoretical and empirical papers and books on schizophrenia, but if it is unfamiliar to neuroscientists then it does not add anything to, and only detracts from, the paper. As an example of how we have edited text to clarify what we mean, we have modified this sentence “On the other hand, it may be the case that within the setting of an overall flattening of response probabilities caused by cortical DA reduction novel associations and meanings can be formed.” so that it now reads as follows: “On the other hand, it may be the case that within the setting of increased randomness in neural activity (e.g., decreased phasic dopamine responses for relevant stimuli and increased spontaneous phasic dopamine release novel associations and meanings can be formed.” We agree with Reviewer 1 that a citation for this point is necessary, and the citation we used is: Maia TV, Frank MJ. An Integrative Perspective on the Role of Dopamine in Schizophrenia. Biol Psychiatry. 2017 Jan 1;81(1):52-66.

*The reader does not care if Cobenfy has a registered trademark.*

***While it is standard in psychiatric journal articles to note when a medication is trademarked, we have removed both the brand name and the trademark in this case, and left only the generic medication name.

*Section 4 is some of the better writing in the paper, but it does not have much relevance to apical amplification concepts. It could be focused.*

***The material in what was Section 4 was moved to Section 2 so that discussion of each neuromodulator and its relevance to apical integration is immediately followed by the evidence for impairment in that neuromodulator system in schizophrenia.

*Similarly, while interesting and well written, the continued discussion of the language symptoms in schizophrenia in this paper are not connected to the central thesis of the work. There is insufficient evidence to link language to their apical integration theory, making the inclusion inappropriate.*

***We acknowledge that this is one of the more speculative sections of the paper. On the other hand, we now note that the nature of the language disturbance in schizophrenia parallels what is seen in the perceptual and memory failures in the disorder, for which it is easier to find links with apical integration (although even here there is no direct evidence in humans). We also emphasize the importance of language disturbance for understanding schizophrenia and the need for further theory building and empirical work on this fundamental symptom. Our goal here is to frame the rationale for testing this hypothesis further, and so we have toned down any language so as to de-emphasize certainty. Note that the Review Editor also suggested that we strengthen this section.

*Section 6: The consciousness argument is not compelling. Using these psychological terms to refer to schizophrenia as a disorder of altered consciousness is not supported by the authors' neurobiological evidence. Do people coming in and out of anesthesia present as schizophrenic? This and section 7 could be deleted.*

***In fact, it is quite common for people coming out of anesthesia to exhibit transient psychotic symptoms such as hallucinations. In the revised manuscript, we better describe the ways in which consciousness is altered in schizophrenia, and we visually depict these in the new Figure 8. Our effort in this section is to describe the ways in which consciousness is disturbed in schizophrenia, since schizophrenia has been described as involving a disturbance in consciousness for over 100 years, even though it has been recognized that it is distinct from (and less severe) than what is seen in delirium and other states of disturbed consciousness. Moreover, profound disturbances of consciousness can occur, including psychotic symptoms, altered sense of body boundaries, uncertainty about one’s existence, depersonalization and derealization, etc. These phenomena have not previously been addressed by theories emphasizing cognitive coordination failures in schizophrenia. We recognize that this section is more speculative than some of the other sections, but we also believe it is important to address “the elephant in the room” which is whether a theory of schizophrenia (or of disorganization in schizophrenia, such as the one we propose) is relevant to some of the most striking clinical features of the disorder. Because we were requested by the Review Editor to keep this section in, we have not deleted it, but we have made every effort to edit this section for clarity, and we have deleted the first paragraph of the section. In addition, we have deleted the entire Section 7, as requested by Reviewer 1.
___________________________________________________________________________________

***Reviewer 2:*** *The manuscript by Silverstein et al is an interesting review which uses the two-point theory to understand disorganization and consciousness in the schizophrenias. Overall, the review demonstrates that the concepts and findings inherent to apical integration theory support the seminal understandings of schizophrenia. In particular, two-point neurons can explain the impairments of integrative functions seen in schizophrenia. The authors propose that a primary factor in the reduction of organized mental activity in schizophrenia is a reduction in the degree to which apical dendrites can modulate processing of input to the soma. This is an extensive and thorough review which may provide further insight into brain dysfunction in schizophrenia. Below are comments which may help improve the clarity of the manuscript:

This review discusses many sub-topics and often reads more like a textbook chapter than a review article. Please consider decreasing the number of different topics discussed and expand on those topics which are considered more relevant. For example, I suggest removing sub-topics which are not extensive (for example, section 3.8 only has two sentences). This will greatly enhance the clarity of the review by highlighting the most relevant information.*

***As noted above, we have shortened the paper by 30% and removed several sections.

*Please reduce the amount of text in 1.2 that describes what each section discusses as this text is redundant.*

***All of this text has been removed. In addition, much of the text from what was section 1.1 has been removed. This reduced redundancy and allows the reader to get to the main points of the paper sooner.

*In Figure 1, the bar graphs in 1A and 1B either need to have a y axis value or included on the same axis (to allow for direct comparison). Otherwise the graphs themselves are uninformative.*

***Figure 1 has been redone in accordance with this suggestion to improve clarity.

*The main take-home message of the review, that two-point neurons can explain the impairments of integrative functions seen in schizophrenia, should be clearly depicted in a Figure. Please either expand upon Figure 1, or have a new Figure.*

***A visual description of the take-home message is provided in Figure 6, which is a revision of the former Figure 4. The revised figure depicts the multiple manifestations of disorganization in schizophrenia as arising from impairments in the function of two-point neurons.

*At times, the text describing biology and cellular properties does not link directly to dysfunctions in schizophrenia. This makes the text disjointed and clarity would be improved if there was always this link involved in every sub-section.*

***In the revised manuscript there are statements about how each biological factor relates to schizophrenia at the end of the paragraph(s) describing that factor. Since there is little evidence from human patients, most of this evidence comes from studies of genetically modified animals and computational studies.

*This review covers two broad main topics; disorganization and consciousness (which could be separated into two different reviews). The link to disrupted consciousness in schizophrenia and two-point neurons is less clear, and this might be enhanced by a dedicated Figure.*

***We have added a new figure (Figure 8) that depicts key aspects of consciousness disturbance in schizophrenia.

____________________________________________________________________________________

***Reviewer 3:*** *Dear editors and authors,

This piece by Silverstein and colleagues has been - genuinely - one of the most intellectually engaging I've had the pleasure to review. Provided my concerns are properly addressed, I will be very happy to recommend acceptance. P.s.: This is a very long manuscript and the deadline for revision was a bit short, so the authors may feel encouraged to underscore any potential inconsistencies in my overall impressions of the manuscript.

Major (essential)
1. Manuscript length and Section 2
My opinion is that section 2 should be radically reduced and simplified. There are already a number of available papers on these topics that the reader may be referred to for better context or further detail. Given the already excessive amount of content we're dealing with in this manuscript, I'd strongly suggest the focus of section 2 to be conveying only what is strictly essential for readers to understand the core ideas of the manuscript. Having read all of it, I fail to see the need for such detailed explanations on topics that - in light of the focus of the actual manuscript (viz. schizophrenia) - may actually deter readers from even starting to read it. This seems especially important given the fact that it's such an introductory section, as it seems to break the natural flow of the manuscript and may substantially undermine its overall impact.
All in all, the idea would be to narrow this section down to the core principles that need to be conveyed, and how they relate to your hypotheses. It'd likely boost clarity and engagement.*

***We have shortened this section and now include statements that refer the reader to major papers that provide elaboration on the issues. In addition, we have integrated the discussion of schizophrenia-related impairments into the relevant sections on neurobiology (e.g., on interneurons, on neuromodulators), and this has eliminated the separation that was present in the first version of the paper between the core neurobiological concepts and anything having to do with schizophrenia. We agree with the reviewer that that separation could have dissuaded readers interested in schizophrenia from even getting to the parts of the paper about the disorder. We assume that some readers of this paper will be neuroscientists who wish to learn about schizophrenia and that others will be schizophrenia researchers who wish to learn more about the hypothesized neurobiology of the disorder. We have tried to balance the presentation of the issues so that both types of readers will be able to update their views.

*2. Biomarkers and robust biological alterations
Any theoretical framework that proposes itself to provide a strong take on the underlying biological mechanisms of schizophrenia/thought disorder should probably be able to accommodate some of its chief and more well-established biomarkers. I found the discussions on some of the foremost ones (e.g., mismatch negativity, prepulse inhibition, gamma-band alterations, parvalbumin interneuron and perineural net alterations, early-ontogeny hippocampal hyperactivity) at times lacking in substance/accuracy (e.g., gamma oscillations are chiefly regulated by PV+ interneurons which carry out perisomatic inhibition, yet the authors at one point seem to speculate these to arise mainly from the apical dendrite) or (potentially) missing altogether. While it's up to the authors which and how many specific biomarkers they'd like to discuss, I strongly recommend discussing some of them in further depth.*

***We wish there was room in the paper for an extended discussion of a range of biomarkers for schizophrenia. Because there is not, however, we have added a few lines of text to note recent reviews and studies covering a wide range of potential markers and endophenotypes in schizophrenia, and we note that our purpose here is mainly to emphasize that a number of these proposed markers are hypothesized to reflect reduced contextual modulation. That is, it is the shared mechanism among many proposed markers, rather than the specific methodologies, that we wish to highlight. We also now indicate that while apical dendrite activity is involved in tuning gamma oscillations, the activity of PV+ neurons plays a greater role in this. And, we mention that some of the mechanisms we discuss (e.g., reduced inhibition) have been proposed to account for additional markers (e.g., motor coordination problems, smooth pursuit eye movement deficits) that have not yet been the focus of apical integration theory. We believe these changes are in the spirit of Reviewer 3’s comment.

*Finally and in the same vein, at various times the authors make speculations on the nature of the pathophysiological alterations in schizophrenia which could be potentially backed - even if indirectly - by hard data on schizophrenia-specific alterations. These may involve cortical microstructure, neurophysiology and whatnot. At times this piece reads overly speculative (which seems natural given the sheer volume of - highly interesting, I should stress - ideas), so it'd be very important to make it sound more falsifiable and empirically-grounded given the extant schizophrenia literature. Suggested literature:
2.1. E/I imbalance and interneurons
Critically, a major concern was the lack of more detailed discussions on E/I imbalance in schizophrenia, especially in what pertains to interneurons. For instance, Parvalbumin+ and Somatostatin+ interneurons, which heavily influence basal and apical compartments of pyramidal cells, have repeatedly been associated with altered function in Sz. These cells play a critical (protagonistic even) role in synchronising the cortical network to gamma and theta oscillatory rhythms (respectively), for example - as well as many of the ionic dynamics underlying the pathophysiological mechanisms you propose. Hence, it seems quite opportune to further explore the modulatory roles of PV+ and SST+ interneurons on apical and basal function. Suggested literature: Almeida and Radanovic, 2022;*

***Thank you for this suggestion. We have now added text on the roles that PV and SST interneurons play in gamma and theta oscillations, and the links between these oscillations and several functions we discuss that are impaired in schizophrenia and that can be viewed as manifestations of reduced contextual modulation. This includes work noting that reduced activity in PV interneurons is thought to be related to excessive excitation and priming in semantic networks (and so is related to formal thought disorder).

*2.2. Role of layers II/III
Layers II/III are one of the most obvious alterations in schizophrenia's cortical structure, yet I'm not sure I recall seeing any mention of this in the manuscript. It is my opinion that this should be either included as a fulcral topic of your model, or alternatively (but less desirably, in my view) adequately addressed as a caveat. I find it unlikely that so many different features of the disease can be explained in real depth if layers II/III are overlooked. Hence, I'd ask the authors: how do you believe layers ii/iii relate to the framework you're proposing?*

*** Because we were trying to cut text to respond to multiple reviewer comments about the length of the paper, we were hesitant to add new sections or new text to the main body of the paper. We believe this is a good question, however, and so we have added the following text to the Discussion section: “A related issue is that this paper focused primarily on integration of apical and basal compartment activity in L5 pyramidal neurons, since much of the evidence for apical integration theory comes from studies of these neurons and they are thus the canonical model for this theory. However, L2/3 pyramidal neurons also have relatively long apical dendrites and are thought to implement contextual modulation. It has also been shown that L2/3 neurons function abnormally in schizophrenia and are characterized by dendritic spine pathology, and these changes are thought to be related to cognitive deficits in the disorder. Integration of dendritic input is less understood in L2/3, however. Moreover, there are structural and functional differences in pyramidal cells in different cortical layers, The similarities and differences in L5 and L2/3 pyramidal cells, in terms of their effects on contextual modulation and its impairments in schizophrenia, is an important area for future work.”

*Minor (irrelevant for acceptance)
1. Congenital blindness: At one point the authors seem to express being somewhat puzzled by the severity of auditory alterations in sz: "Impairments in both visual and auditory
selective attention have been demonstrated in schizophrenia, although, interestingly, these appear to be more severe in the auditory domain [129,130].".
I'd suggest looking into literature about congenital blindness and schizophrenia. If nothing else, it may be very informative: there is a striking scarcity of reported cases of congenitally blind individuals who have developed SZ. This is quite likely to involve compensatory plasticity of the auditory cortex during early development, which in turn should enhance auditory discrimination (e.g., MMN amplitudes) as well as aspects of language function which are directly related to thought disorder, inter alia. What thoughts do the authors have on this?*

***The first author (SMS) wrote one of the first reviews of this fascinating literature: Silverstein SM, Wang Y, Keane BP. Cognitive and neuroplasticity mechanisms by which congenital or early blindness may confer a protective effect against schizophrenia. Front Psychol. 2013 Jan 21;3:624. We also wrote a brief follow-up: Silverstein SM, Wang Y, Roché MW. Base rates, blindness, and schizophrenia. Front Psychol. 2013 Apr 3;4:157. doi: 10.3389/fpsyg.2013.00157. As it turns out, we have removed the statement about selective attention impairments possibly being more severe in the auditory domain in schizophrenia. While it is what the literature says, it was deleted as part of the overall effort to cut text that was less relevant to the paper’s goals. This is one of those cases then where “if we only had more room…….”

*2. 5HT2A receptors are at one point mentioned. Cortically, these are densely expressed on the apical dendrites of layer Vb pyramids - yet, they noticeably have a depolarising effect. It would be useful for the authors to better clarify this relationship and how it might or not endorse their model.*

***Thank you for this suggestion. We have made several changes in response. First, we now better integrate the discussions of neuromodulators with discussions of evidence for altered neuromodulation in schizophrenia. Second, we have added text to the discussion of each neuromodulator in schizophrenia to clarify how abnormalities in each system could give rise to specific symptoms. Third, for serotonin and also other modulators, we have clarified how the evidence in schizophrenia suggests that there may be a general landscape of reduced contextual modulation, but one in which there can be isolated regions of excessive apical activity or apical drive, which could lead to both disorganized and positive symptoms, respectively, and to increased disconnection from external reality over time.

*3. The authors have underscored disintegration of perceptual and other representations in schizophrenia. In vision, SST+ interneurons seem to provide widespread divisive inhibition (eg surround suppression) which can synchronise large ensembles of neurons in a way that may be congruent with the establishment of this "unity" - basically by modulating the activity of apical dendrites of multiple pyramids, radially. Might SST+ hypofunction be related to this effect in schizophrenia?*

***This is an interesting suggestion. Ultimately, because we deleted the section on surround suppression to save space, it did not make sense to elaborate on the issue of divisive inhibition (i.e., the extent to which apical amplification mechanisms might be involved in suppressive effects). Others (e.g., Phillips) have noted that this is an area needing further study. We agree with that, and have decided that there was not room in the current manuscript to address those issues.

*4. The authors mention reduced drive to the apical dendrite. However, there is also reduced inhibition. Might this hint at a reduced overall dynamic range of apical amplification/suppression in schizophrenia, and hence a more inflexible (and thus context-insensitive) state of weak disinhibition of apical dendrites?*

***Thank you for this suggestion. We have added this idea to the section on interneurons and their pathology in schizophrenia.

*5. Intrusive memories: Almeida (2024) proposes that the disinhibition of apical dendrites from SST+ influence is most likely the core reason driving mnemonic interference/intrusions, which seems to directly relate to some of your claims on intrusive memories in SZ. Any thoughts on this?*

***We agree with your thinking on this. We now note in the section on interneurons that SST interneurons have the function of reducing interference between neural ensembles and that weakening of SST-IN activity is related to intrusion of memory traces in Alzheimer’s disease (and we cite Almeida, 2024). We then note that: “This is relevant to schizophrenia as well, however, because, as we describe in Section 3, it is characterized by both reduced SST activity and increased intrusions in perception, memory, and language.” We then return briefly to the issue of SST interneuron impairment when discussing memory intrusions in schizophrenia later in the paper, and note how this may be similar to what is happening in perception and language.

*Key takeaway to the authors and editor

My assessment is that this manuscript is in fact highly valuable, though I believe it might need polishing - especially in terms of further discussion and integration of real-world, schizophrenia-specific data (e.g., cortical microstructure, neuroanatomy, electrophysiological biomarkers, current models etc). These more robust findings should ideally be discussed and integrated into any theoretically bold and useful model of schizophrenia - after all, the model has to accomodate the data. Altogether and to put it more plainly: at times it felt like the content gravitates more into discussing DIT than the pathophysiology of schizophrenia/thought disorder itself (e.g., as per my comments on the lack of discussion of critical aspects of the disaese, such as like mismatch negativity, layers II/III, neuroanatomical data, PV+ disinhibition and perineural nets etc, whereas anything DIT-related appeared to be discussed in far greater detail).*

***We appreciate your helpful feedback on the paper. We have made every effort to improve the arguments in the paper by better integrating the biological and clinical data, and by adding additional evidence from schizophrenia research. This reduces the previous split between the discussion of apical integration theory and the discussion of manifestations of disorganization in schizophrenia, although the sequence of the presentation of the biological mechanisms (and, now, their impairments in schizophrenia) followed by the clinical and laboratory manifestations of disorganization in schizophrenia still remains.

*References (potentially useful, fully optional)*

***Thank you for providing these references. We have included most of them in the revised version of the manuscript.

*Palaniyappan, L., Homan, P., & Alonso-Sanchez, M. F. (2023). Language network dysfunction and formal thought disorder in schizophrenia. Schizophrenia Bulletin, 49(2), 486-497.
Almeida, V. N. (2024). Somatostatin and the pathophysiology of Alzheimer's disease. Ageing Research Reviews, 96, 102270.
Almeida, V. N., & Radanovic, M. (2021). Semantic priming and neurobiology in schizophrenia: A theoretical review. Neuropsychologia, 163, 108058.
Morgan, V. A., Clark, M., Crewe, J., Valuri, G., Mackey, D. A., Badcock, J. C., & Jablensky, A. (2018). Congenital blindness is protective for schizophrenia and other psychotic illness. A whole-population study. Schizophrenia research, 202, 414-416.
Javitt, D. C., Lee, M., Kantrowitz, J. T., & Martinez, A. (2018). Mismatch negativity as a biomarker of theta band oscillatory dysfunction in schizophrenia. Schizophrenia research, 191, 51-60.
Lakatos, P., O'Connell, M. N., Barczak, A., McGinnis, T., Neymotin, S., Schroeder, C. E., ... & Javitt, D. C. (2020). The thalamocortical circuit of auditory mismatch negativity. Biological psychiatry, 87(8), 770-780.
Javitt, D. C., Steinschneider, M., Schroeder, C. E., & Arezzo, J. C. (1996). Role of cortical N-methyl-D-aspartate receptors in auditory sensory memory and mismatch negativity generation: implications for schizophrenia. Proceedings of the National Academy of Sciences, 93(21), 11962-11967.
Steullet, P., Cabungcal, J. H., Coyle, J., Didriksen, M., Gill, K., Grace, A. A., ... & Do, K. (2017). Oxidative stress-driven parvalbumin interneuron impairment as a common mechanism in models of schizophrenia. Molecular psychiatry, 22(7), 936-943.
Santos-Silva, T., dos Santos Fabris, D., de Oliveira, C. L., Guimarães, F. S., & Gomes, F. V. (2024). Prefrontal and hippocampal parvalbumin interneurons in animal models for schizophrenia: a systematic review and meta-analysis. Schizophrenia Bulletin, 50(1), 210-223.*

Accept Letter

Dear Dr. Silverstein,   
 
Thank you for submitting your manuscript to Current Research in Neurobiology.

I am pleased to inform you that your manuscript has been accepted for publication. Congratulations.
    
Your accepted manuscript will now be transferred to our production department. We will create a proof which you will be asked to check, and you will also be asked to complete a number of online forms required for publication. If we need additional information from you during the production process, we will contact you directly.

We appreciate and value your contribution to Current Research in Neurobiology. We regularly invite authors of recently published manuscript to participate in the peer review process. If you were not already part of the journal’s reviewer pool, you have now been added to it. We look forward to your continued participation in our journal, and we hope you will consider us again for future submissions.

*CRNEUR* aims to be a unique, community-led journal, as highlighted in the [Editorial Introduction](https://www.sciencedirect.com/science/article/pii/S2665945X21000012). As part of this vision, we will be regularly seeking input from the scientific community and encourage you and your co-authors to take the [survey](https://www.surveymonkey.co.uk/r/5LHWTML).

We would also like to invite you to take part in our CRNEUR Author [Question & Answer (Q&A)](https://www.elsevier.com/__data/promis_misc/QA_Authors.docx), which could get published alongside your article and help to promote it. We suspect you might have an interesting story of perseverance or team work that was required for the research study to complete, or a diversity of perspectives that you might share, as a way of inspiring others about neuroscience.

Kind regards,

Anna S Mitchell, Ph.D.  
Editor in Chief  
Current Research in Neurobiology  
  
and Guest Editor Professor William Phillips

*-------- End of Review Comments --------*
